# Community phylogenetics at the biogeographical scale: cold tolerance, niche conservatism and the structure of North American forests

**DOI:** 10.1111/jbi.12171

**Published:** 2013-07-31

**Authors:** Bradford A Hawkins, Marta Rueda, Thiago F Rangel, Richard Field, José Alexandre F Diniz-Filho, Peter Linder

**Affiliations:** 1 Department of Ecology & Evolutionary Biology, University of CaliforniaIrvine, California, USA; 2 Departamento de Ecologia, ICB, Universidade Federal de GoiásGoiânia, Goiás, Brazil; 3 School of Geography, University of NottinghamNottingham, UK

**Keywords:** Forest phylogenetics, National Forest Inventory, niche conservatism, North America, phylogenetic signal representation, random forests, trait evolution, tree communities, tropical conservatism hypothesis

## Abstract

**Aim**The fossil record has led to a historical explanation for forest diversity gradients within the cool parts of the Northern Hemisphere, founded on a limited ability of woody angiosperm clades to adapt to mid-Tertiary cooling. We tested four predictions of how this should be manifested in the phylogenetic structure of 91,340 communities: (1) forests to the north should comprise species from younger clades (families) than forests to the south; (2) average cold tolerance at a local site should be associated with the mean family age (MFA) of species; (3) minimum temperature should account for MFA better than alternative environmental variables; and (4) traits associated with survival in cold climates should evolve under a niche conservatism constraint.

**Location**The contiguous United States.

**Methods**We extracted angiosperms from the US Forest Service's Forest Inventory and Analysis database. MFA was calculated by assigning age of the family to which each species belongs and averaging across the species in each community. We developed a phylogeny to identify phylogenetic signal in five traits: realized cold tolerance, seed size, seed dispersal mode, leaf phenology and height. Phylogenetic signal representation curves and phylogenetic generalized least squares were used to compare patterns of trait evolution against Brownian motion. Eleven predictors structured at broad or local scales were generated to explore relationships between environment and MFA using random forest and general linear models.

**Results**Consistent with predictions, (1) southern communities comprise angiosperm species from older families than northern communities, (2) cold tolerance is the trait most strongly associated with local MFA, (3) minimum temperature in the coldest month is the environmental variable that best describes MFA, broad-scale variables being much stronger correlates than local-scale variables, and (4) the phylogenetic structures of cold tolerance and at least one other trait associated with survivorship in cold climates indicate niche conservatism.

**Main conclusions**Tropical niche conservatism in the face of long-term climate change, probably initiated in the Late Cretaceous associated with the rise of the Rocky Mountains, is a strong driver of the phylogenetic structure of the angiosperm component of forest communities across the USA. However, local deterministic and/or stochastic processes account for perhaps a quarter of the variation in the MFA of local communities.

## INTRODUCTION

At the biogeographical scale the evolution of cold tolerance represents a key innovation that has permitted many tree clades to persist in high northern latitudes after the global cooling initiated at the end of the Eocene (Latham & Ricklefs, 1993; Ricklefs, 2005; Donoghue, 2008). The fossil record for North American, Asian and European trees is broadly consistent with this (Hsu, 1983; Latham & Ricklefs, 1993; Graham, 1999; Ricklefs, 2005), woody clades have undergone relatively slow rates of climatic niche evolution over long periods of time (Smith & Beaulieu, 2009), and angiosperm families comprising trees become progressively younger on average moving from the equator northwards (Hawkins *et al*., 2011) – a phylogenetically structured spatial pattern measured by mean family age. Thus, multiple lines of evidence indicate that for angiosperms at least, geographical patterns of tree diversity are consistent with the tropical conservatism hypothesis (Wiens & Donoghue, 2004). Under that hypothesis phylogenetic niche conservatism (PNC) of ancestral traits associated with tropical conditions has largely driven the phylogenetic structure and composition of regional species pools, where minimum temperatures to which regions are exposed determine which clades are able to persist in them. And although niche evolution has obviously occurred in multiple tree clades throughout their history, the evolution of cold tolerance among trees has been difficult and has come at a cost, with a trade-off between freezing tolerance and growth rates in warm climates (Koehler *et al*., 2012).

Local communities are necessarily assembled from regional species pools: if we can assume that cold tolerance is the primary determinant of which clades of trees occur in different parts of North America and that niche conservatism in trees is strong and manifested at the family rank, then we can predict that the continental-scale pattern of family age structure will be apparent at the local scale. (See Latham & Ricklefs, 1993, for fossil-based evidence that cold tolerance is conserved at the family rank and that entire tree families have responded to mid-Tertiary climate change.) That is, local forest communities will comprise species from older families carrying the ancestral trait (intolerance to freezing) in warmer climates and species from younger families carrying the derived trait (freezing tolerance) in colder climates (prediction 1). This should be true above and beyond any other influences on community composition. Second, although many traits influence the assembly of forest communities and facilitate species coexistence (see e.g. Swenson & Weiser, 2010; Koehler *et al*., 2012; Kunstler *et al*., 2012; Lines *et al*., 2012; Swenson *et al*., 2012; Drescher & Thomas, 2013), cold tolerance should be strongly associated with the mean family age of local communities relative to other traits (prediction 2). Third, minimum temperatures to which local communities are exposed should be the primary environmental correlate of mean family age in local communities of angiosperm species (prediction 3), even if other climatic influences on trees also exist (Sakai & Weiser, 1973).

An important aspect of evaluations of tropical niche conservatism as an explanation for tree phylogenetic composition at either biogeographical or local scales is the assumption that cold tolerance is phylogenetically conserved. The fossil record provides evidence that cold tolerance contains phylogenetic signal (see e.g. Latham & Ricklefs, 1993), but by some definitions that is not sufficient to assume PNC, which Losos (2008, p. 996) defines as ‘the phenomenon that closely related species are *more* ecologically similar than might be expected solely as a Brownian motion evolution’. Although strong PNC can also result in an apparent lack of phylogenetic signal (Wiens *et al*., 2010), and for other workers Brownian motion evolution is sufficient to define PNC, it remains that phylogenetic conservatism of traits of interest must exist if it is to be invoked as an explanation for observed patterns, whether for cold tolerance or any other presumed key trait (prediction 4). We agree with Losos (2008) that PNC should be demonstrated empirically, if possible, irrespective of disagreements about whether a Brownian motion model of trait evolution is sufficient or not (see also Cooper *et al*., 2010, for discussion of alternative macroevolutionary models underlying PNC).

In this study we tested the four predictions using two sets of analyses: (1) geographical analysis of local community data from the US Forest Service's Forest Inventory and Analysis (FIA) database; and (2) macroevolutionary analyses of trait evolution. Using the spatial FIA data set, we documented the geographical pattern of mean family age (MFA) of angiosperm communities to test whether the biogeographical pattern of decreasing family age to the north identified by Hawkins *et al*. (2011) generates a similar pattern at the local scale (prediction 1). This is the metric of phylogenetic structure that we attempt to understand, because it provides a core measure of tropical niche conservatism for North American trees (Latham & Ricklefs, 1993). This metric differs from the phylogenetic dispersion metrics normally associated with the field of ‘community phylogenetics’, but we feel that the field can be broadened to include other aspects of phylogenetically structured community composition, especially linking traits to patterns of community composition analysed in an explicit phylogenetic framework.

Our macroevolutionary analyses were of five traits for which we could obtain information for at least 60% of species and which we felt could be physiologically linked to mean family age, including an estimate of cold tolerance, seed size, dispersal mode, leaf phenology and height, to test for PNC (prediction 4). Using the FIA data, we then evaluated the relationships between mean trait values in local communities and MFA to identify the traits most strongly associated with phylogenetic structure (i.e. MFA) (prediction 2). Finally, we examined 11 environmental variables operating at broad and/or local scales to determine whether minimum winter temperature best explains statistically the geographical pattern of MFA in the FIA data (prediction 3). It was not our goal to identify all of the local and regional influences on community structure, although local communities are influenced by both (e.g. White & Hurlbert, 2010; Lessard *et al*., 2012). Rather, we evaluated the extent to which cold tolerance and other traits that facilitate survivorship in cold climates are phylogenetically conserved, and the extent to which tropical niche conservatism can account for the age structure of local arboreal angiosperm communities.

## MATERIALS AND METHODS

### Forest community data

The community data comprise 91,340 plots, each of 0.07 hectares, in the contiguous USA extracted from the US Forest Service's Forest Inventory and Analysis (FIA) database (http://www.fia.fs.fed.us/, accessed in January, 2012). For inclusion, a site had to support at least two angiosperm tree species and be coded as a ‘natural stand’. All gymnosperms were removed from the data because they have very different evolutionary histories from angiosperms (Graham, 1999). Sites from Alaska were also excluded because they are primarily or exclusively composed of gymnosperms.

### Tree phylogeny

**Figure 1:**
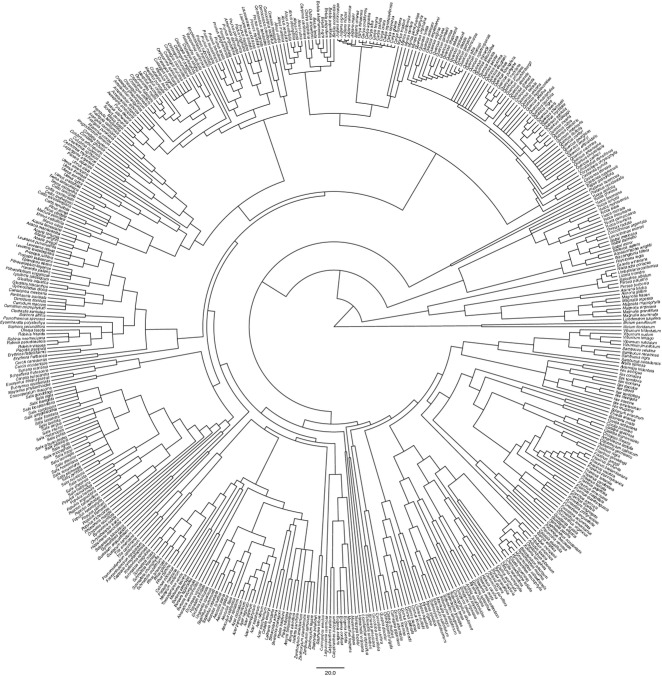
Phylogeny for 500 North American angiosperm tree species. Branch lengths are in millions of years. See high resolution linear version of Fig. 1 in Appendix S2 and Newick version in Appendix S1.

The phylogeny used to examine trait evolution was based on APG III (Angiosperm Phylogeny Group, 2009), to which we added lower taxonomic ranks using the mega-phylogeny of Smith *et al*. (2011) (available at http://datadryad.org/resource/doi:10.5061/dryad.8790) and group-specific phylogenies available in the primary literature (Appendix S1 in Supporting Information). All species of angiosperms were included in the phylogeny, whether sampled or not by the FIA (Fig. 1; high resolution linear version in Appendix S2 and Newick version in Appendix S1). The definition of trees was that of Elias (1980), which includes some groups and species not universally considered to be trees (e.g. some yuccas and cacti), and we included all currently recognized species native to North America north of Mexico, irrespective of whether or not they were sampled by the FIA. Infra-familial tree structure was based on the most highly resolved group-specific phylogenies we were able to locate. When it was not possible to determine the most likely phylogenetic relationships, judging by the conclusions of the original authors or inconsistencies between genes or studies, we treated the relevant taxa as polytomies. No branch lengths were included. Species not resolved or not included in the phylogenies were treated as basal polytomies. We calibrated the phylogeny by dating nodes with the branch length adjustment function (BLADJ) in phylocom (Webb *et al*., 2008), using 74 nodes in our phylogeny matched against the dated phylogeny of Bell *et al*. (2010), which represents the most complete source for node ages of angiosperms although many of their family age estimates are less accurate than alternative sources of information (see next section). BLADJ assigns undated nodes equal branch lengths between nodes for which age estimates are available.

### Mean family age (MFA)

We used two estimates of family crown ages to generate MFA. First, we use the molecular-based ages provided by Davies *et al*. (2004). MFA was calculated by assigning all species the age of the family to which they belong (see Appendix S3) and then averaging across all species occurring at each site. We used Davies *et al*. (2004) rather than Bell *et al*. (2010) for the geographical analysis because a comparison of their estimates against fossil-based age estimates for the families sampled by the FIA indicated that the ages in Davies *et al*. (2004) more closely matched the fossil record than did the ages of Bell *et al*. (2010) (Appendix S3), suggesting that there are some potential problems with the age estimates in the later study. Because we were able to obtain fossil-based minimum ages for all but three families (comprising 10 species) sampled by the FIA, we also generated MFA based on these ages, using mid-points when age estimates were bounded or minimum age + 1 Myr when only minimum bounds were known. This allowed us to evaluate the robustness of the MFA pattern with respect to source of the age estimates, although we used the more complete and arguably more precise data from Davies *et al*. (2004) for analytical purposes.

The use of family to assign ages assumes that there is strong conservation of traits relevant to geographical distributions at the family rank, an assumption for which there is fossil-based support (Latham & Ricklefs, 1993). It also ignores intra-familial variation in traits, which if extensive could obscure broader patterns originating from patterns of trait evolution deep in the history of angiosperms. Like all taxonomic ranks above species, family designations contain arbitrary components, but using distribution maps for families and their ages Hawkins *et al*. (2011) found a strong latitudinal gradient of mean age of arboreal families in North America consistent with a tropical conservatism prediction. This indicates that interpretable evolutionary signal is contained at the family level, at least for arborescent angiosperms. Calculating MFA also permits a direct comparison of the local patterns generated at the species level with that found by Hawkins *et al*. (2011) using range maps of families and a global grid system. It is also important to realize that ages of lower ranks (particularly species) are not appropriate for the type of analysis we are conducting, for two reasons. First, species ages depend on diversification rates more than niche conservatism and thus address a different mechanism. Second, ages of lower taxonomic ranks are expected to be more biased by extinctions than higher ranks, which can make ages of surviving taxa appear much older than they are; many more species will have gone extinct in the Cenozoic than entire families. Although family level is not ideal for the reasons already mentioned, it represents the best available option for evaluating conservatism of traits originating deeper in the tree phylogeny.

### Traits

We selected five traits for which data could be obtained for more than 60% of the North American angiosperm trees and which could plausibly be physiologically and phylogenetically linked to family age via PNC – and thus could offer causal explanations for observed gradients. These were realized cold tolerance, seed size (log_10_ transformed), seed dispersal mode (ranked), leaf phenology (categorized) and normal maximum height (see Appendix S3 for trait values). These traits were used directly for cross-species comparative analyses and averaged across the species within each site for geographical analyses and associations with environmental data. Trait values were assigned at the species level, which ignores potential local adaptation at the population level, but complete intra-specific information across the USA does not exist.

**Figure 2:**
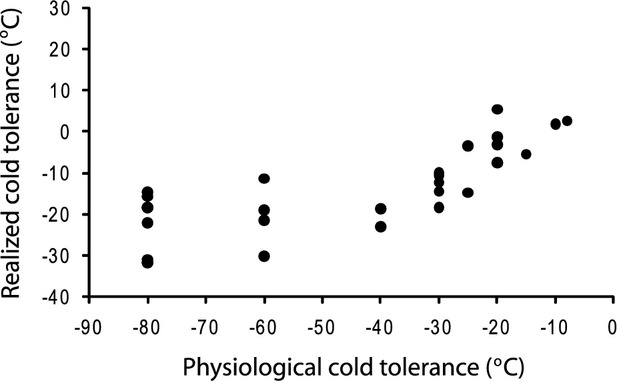
Relationship between realized cold tolerances (minimum temperature experienced within current distribution) and physiological cold tolerances estimated by Sakai & Weiser (1973) for 30 North American angiosperm tree species.

Cold tolerance represents a key trait under the tropical conservatism explanation for tree distributions. Physiological cold tolerances are not known for all species, so we generated ‘realized’ tolerances by overlaying species range maps derived from Elias (1980) on the BIO6 raster (minimum temperature in the coldest month) from the 10 arc-minute WorldClim database (available at http://www.worldclim.org/) and recording the coldest temperature within each range. This is unlikely to represent the physiological cold tolerances of most trees, but we validated the assumption that minimum temperatures experienced by each species are positively associated with physiological tolerances by comparing realized cold tolerances with experimentally determined cold tolerances for the 30 species of angiosperms tested by Sakai & Weiser (1973). There was a reasonably strong relationship across all species (*r *=* *0.773, Fig. 2), but it is nonlinear because the realized cold tolerances cannot exceed −40 °C: the minimum temperature in the parts of North America capable of supporting any trees. This also indicates that range edges of angiosperms in northern Canada and Alaska may be set by more than minimum temperatures, probably also being limited by the short growing season and permafrost soils. When species with physiological cold tolerances lower than they could experience in North America were removed, the correlation between realized and actual cold tolerances improved to *r *=* *0.834. Thus, realized cold tolerance appears to provide a reasonable measure of the relative cold tolerances of trees currently growing on the continent, particularly in the contiguous United States. We generated cold tolerance values for all 500 angiosperms in the phylogeny (for phylogenetic comparative analysis) and all 212 species in the FIA data (for geographical analysis).

We also assigned normal maximum heights for all species in both data sets using Elias (1980); we would expect average heights of angiosperms to be lower to the north and west because trees are shorter when growing in cold or arid climates (Lines *et al*., 2012). Seed size, known to decrease in the Northern Hemisphere from south to north (Moles *et al*., 2007) was obtained for 321 (64%) of the species occurring in North America and 171 (81%) of the species in the FIA data set. Seed dispersal mode (unassisted, animal or wind, with 10 water-dispersed species excluded) was obtained for 479 (96%) North American species and 205 (97%) of the FIA species, and leaf phenology (evergreen versus deciduous) was obtained for all species. Seed size and dispersal mode are likely to covary and be spatially structured, perhaps because of fewer species of animal seed dispersers to the north, stronger winds in more open habitats, or the faster spread of small, wind-dispersed seeds as trees recolonized northern North America after the Last Glacial Maximum. The evolution of deciduousness is likely to be at least partially linked to cold tolerance, because it has permitted trees to colonize seasonally cold or dry areas.

We extracted the data for seed size and dispersal mode primarily from the Kew Seed Information Database (http://data.kew.org/sid/, accessed in February 2012). The Kew SID gives the dry mass of 1000 seeds for each species. However, the headline statistic retrieved by a search on any given species can be problematic, for instance because it is the mean of data that include fresh mass. Therefore, for each species we examined the data behind the headline statistic, excluding problematic values and recalculating the mean when necessary (performed for 120 of 1656 individual values, including values for gymnosperms that have been excluded from this analysis). Some species were not listed in the Kew SID, or had no data for seed mass or dispersal mode. For these we searched other sources for information in scientific papers, the USDA Plants Database (http://plants.usda.gov/java/) and the University of Texas at Austin's Native Plant Database (http://www.wildflower.org/plants/). For a few species we assigned dispersal mode using information for congenerics [e.g. for some oaks (*Quercus*) and walnuts (*Juglans*)], in conjunction with photographic evidence from diaspores and/or personal knowledge of the trees. The basis for assigning dispersal mode was the distance seeds were likely to be dispersed; very light seeds carried by wind (> 1 km dispersal distances) were classified as wind dispersed, seeds carried and/or eaten by birds and mammals were considered medium dispersers (50 m to 1 km dispersal distance) and were classified as animal dispersed, and heavy seeds (whether winged or not) not known to be dispersed by animals were classified as unassisted (< 50 m dispersal distances). We obtained leaf phenology data from the TRY database (http://www.try-db.org/, accessed in February 2012; Kattge *et al*., 2011), again supplemented with data from the sources also used for seed size. Several species had data under different names; we used The Plant List (http://www.theplantlist.org/) as the source of information on synonyms.

### Environmental data

We selected 11 variables to examine the relationship between environment and the geographical structure of mean family age across sites. Seven represent factors varying over broad and intermediate scales while simultaneously, because of the methods by which they were generated, having little or no variation at local scales. We extracted two measures of temperature and three of precipitation from the 30 arc-second WorldClim database: BIO5 (maximum temperature of the warmest month), BIO6 (minimum temperature of the coldest month), BIO12 (annual precipitation), BIO9 (precipitation in the driest quarter) and BIO18 (precipitation in the warmest quarter). The expectation is that minimum temperatures should be the variable most strongly associated with mean family age at the continental extent. We also calculated summer soil moisture, derived from the European Space Agency global soil moisture data set (http://www.esa-soilmoisture-cci.org/, accessed in June 2012). Daily soil moistures were averaged over 1 June to 31 August over five haphazardly selected years with good coverage of North America (1983, 1995, 2000, 2005 and 2008). Although this variable contains moderate patchiness at intermediate scales, the grain of computation (*c*. 25 km) makes it unsuitable for examining local-scale variation. Finally, we classified sites as being ice free or glaciated during the Last Glacial Maximum, using the 18 ka map of Dyke *et al*. (2003). The local communities at a minimum of 30,437 of the sites in our analysis had to be assembled through primary succession as the ice retreated.

We also generated four variables containing small-scale variation to represent proxies for potential local processes. We estimated the elevation of each site using the digital elevation model gtopo30 (http://www1.gsi.go.jp/geowww/globalmap-gsi/gtopo30/gtopo30.html), although this method generates some error because the Forest Service shifts the geographical coordinates of some sites slightly to protect the privacy of private landowners. We extracted site slope and aspect directly from the FIA database. Finally, we recorded the categorical physiographic class code (PHYSCLCD), also from the FIA, defined as ‘The general effect of land form, topographical position, and soil on moisture available to trees’ (http://fia.fs.fed.us/library/database-documentation/, version 5.1 for Phase 2, p. 60). Although this highly local variable might be expected to co-vary with the coarser, remotely sensed ESA soil moisture, an ANOVA indicated no relationship between them (model *r*^2^ = 0.003).

### Analytical protocols

#### Phylogenetic comparative analyses (for testing prediction 4)

We used multiple approaches to evaluate evolutionary patterns in the traits. First, we examined the phylogenetic signal of each tree trait using a phylogenetic signal representation curve (PSR) approach (Diniz-Filho *et al*., 2012), which is derived from phylogenetic eigenvector regression (PVR; Diniz-Filho *et al*., 1998). In PVR, selected eigenvectors extracted from a phylogenetic distance matrix are used to model interspecific variation for a trait. In PSR, sequential PVR models are fitted after successively increasing the number of eigenvectors and plotting their *R*^*2*^ against the accumulated eigenvalues extracted from the phylogenetic distance matrix. Diniz-Filho *et al*. (2012) demonstrated that under a Brownian motion model of evolution for quantitative traits the relationship between the *R*^*2*^ values of the PVRs and the cumulative eigenvalues is linear, and the pattern of the deviations from linearity reflects alternative evolutionary models. The PSR area, expressing deviations from Brownian motion across the curve, is strongly correlated with Blomberg's *K* statistic, so nonlinear PSR curves reveal whether traits are evolving at a slower or faster rate than expected under Brownian motion in different parts of the phylogeny (expressed by the location of deviations along the eigenvalue axis). For example, in an Ornstein–Uhlenbeck (OU) process, the PSR curve lies below the Brownian linear expectation, and the PSR area is correlated with the strength of the OU process (the α parameter, which can also be expressed as phylogenetic half-life). Thus, PSR provides an elegant exploratory method for understanding deviations from Brownian motion in terms of acceleration or deceleration of evolutionary rates at large or small phylogenetic distances. We used the pvr package in R (see http://cran.r-project.org/web/packages/PVR/index.html) for calculating the PSR curves for each quantitative species trait.

We also followed Kozak & Wiens (2010) and Wiens *et al*. (2010) and tested for niche conservatism in each trait using the general approach of Butler & King (2004). We calculated the log-likelihood of phylogenetic generalized least squares (pGLS) fitting of three models of evolution for each species trait: a white noise (WN) model of random variation, in which the similarity of species is independent of their phylogenetic relationships; a Brownian motion (BM) model of gradual and continuous drift in species' traits (sufficient to demonstrate PNC according to one definition); and an Ornstein–Uhlenbeck (OU) model of constrained evolution (i.e. stasis or stabilizing selection – see Hansen & Martins, 1996; Hansen, 1997; Hansen *et al*., 2008), which satisfies the most stringent definition of PNC (Losos, 2008). The first two models (WN and BM) do not explicitly incorporate constraints in evolution, and the covariance among species will be independent (WN) or linearly associated with phylogenetic relatedness (BM). The OU model, in contrast, describes constrained character evolution in which traits are ‘pulled’ towards an optimum value (Kozak & Wiens, 2010), and even if the restraining force (α) is strong enough to eliminate all phylogenetic signal, the variance of the trait will be much smaller than expected under BM or WN models (although the latter is difficult to assess in practice because there is no explicit expectation of trait variance under alternative evolutionary processes – see Revell *et al*., 2008). We used geiger in R (Harmon *et al*., 2008) to calculate the log-likelihood of each model and compared the fit of each model using a likelihood test, converging to a chi-square distribution, to determine whether each trait fits an OU model better than either a BM or WN model. Weights of the Akaike information criterion (AIC) derived from model likelihoods were also used as an alternative approach to evaluate the best-fitting model (and generated slightly different results).

Both PSR and fitting models using pGLS are designed for quantitative traits, and expectations for discrete traits may differ. So, for leaf phenology and seed dispersal mode we used a simulation approach to obtain the distribution of expected values of PSR area and the expected difference between likelihoods of pGLS models. The observed values of PSR area and likelihood differences were compared with an empirical distribution of values obtained by simulating 250 Brownian motion processes on the phylogeny using geiger. The simulated continuous traits were then transformed into a discrete trait keeping the observed frequency of the original trait (Fritz & Purvis, 2010) and then analysed using the PSR curves and pGLS model fits for each simulation.

#### Geographical analysis (for testing predictions 1–3)

Analysis of mean family age also followed several steps. First, four random forest models were generated in Statistica 8.0 to account statistically for MFA across sites, one model including only traits, one including broad-scale environmental predictors only, one including local-scale environmental predictors only, and a combined model including all predictors. One hundred regression trees were generated in each run, and variable importance values and test risk estimates (squared error rates) were recorded. Thus, the first random forest model identified the most important traits (as defined by the method and available traits), the second identified the most important broad-scale environmental predictors, the third identified the most important local-scale environmental predictor, and the fourth ranked the simultaneous contributions of all variables to MFA. Potential collinearity among predictors within each model can be indicated by relative importance values from random forest models, but it also was evaluated with general linear modelling. We first generated linear models that included the most important predictor variable (environmental or trait) identified by the random forest models and compared those models against models in which all environmental, trait or combined variables were included. Increases in the coefficient of determination represent the contribution of the remaining variables not collinear with the most important predictor.

#### Spatial evaluation (for testing predictions 2–3)

We evaluated the ability of the predictor variables to account for the spatial structure of mean family age using spatial correlograms generated in sam 4.0 (Rangel *et al*., 2010). Because of computational limitations a correlogram for MFA was generated based on 15,000 randomly selected sites. We then generated a correlogram for the same subset of sites using the residuals of the combined random forest model generated using all sites. The difference between the correlograms quantifies the amounts of spatial pattern explained by the model across scales and identifies any scales at which the model is unable to fully explain the spatial pattern (Diniz-Filho *et al*., 2003).

## Results

**Figure 3:**
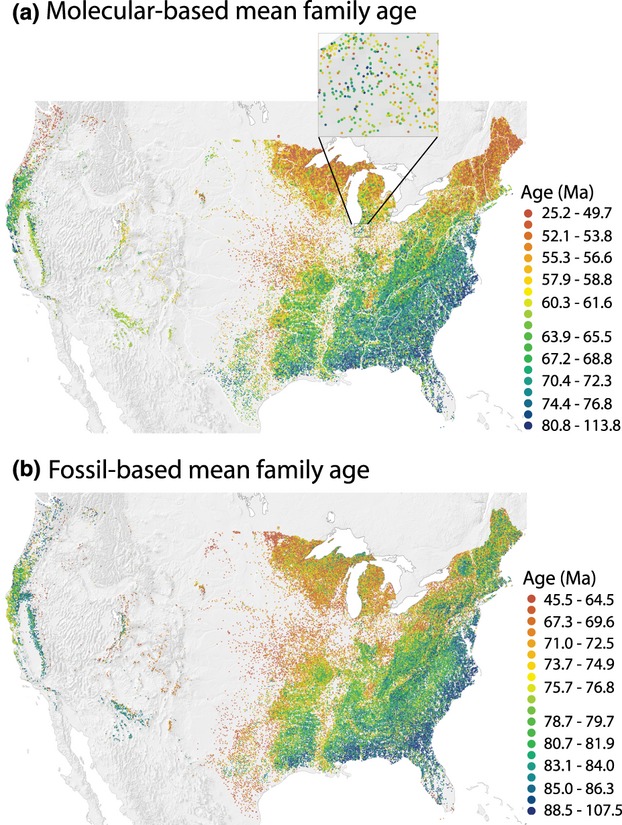
Geographical pattern of mean family age for North American angiosperm tree species across 91,340 plots based on (a) molecular dates from Davies *et al*. (2004), and (b) fossil dates. Major rivers are shown in white. The insert in (a) exemplifies variation at local scales.

Mean family age shows geographical pattern across the contiguous USA at almost all spatial scales whether using molecular-or fossil-based ages (Fig. 3). Focusing on the molecular-based results (Fig. 3a), at the larger scale there is a striking ‘latitudinal’ gradient, with the angiosperm component of forest communities dominated by trees from older families in southern forests and from younger families to the north. This is consistent among eastern, western-montane and west-coast forests, although southern montane forests tend to be younger than lowland forests at equivalent latitudes. There are also regional longitudinal gradients, especially in the eastern forests, with lowland forests nearer the east coast comprising older families than forests at the forest–prairie interface. There is no evidence of a longitudinal gradient at the largest scale across the entire USA, because both eastern and western forests show a pattern of older MFAs near the coast in the south but younger ages to the north (Fig. 3a). Mean fossil-based ages differ in several respects (Fig. 3b), but many of the age estimates are imprecise given the current state of the fossil record. Most notable is that average ages were substantially older in most places than those using molecular-based family ages.

A few other patterns stand out at intermediate scales. For example, Appalachian forests tend to be from younger families than surrounding lowland sites, and forests along the lower Mississippi River tend to comprise species from younger families than other southern forests to the east and west for both age estimates (Fig. 3). Additional smaller-scale patches also exist in various areas without obvious associated geographical features. Finally, patchiness occurs down to the smallest scale of the data, although there is also substantial variation at very local scales: sites within a few kilometres of each other can have very different mean family ages (Fig. 3a insert). The structure of the data strongly implies that both biogeographical and local factors influence the phylogenetic structure of angiosperm tree communities across the central section of the continent represented by the contiguous USA.

**Figure 4:**
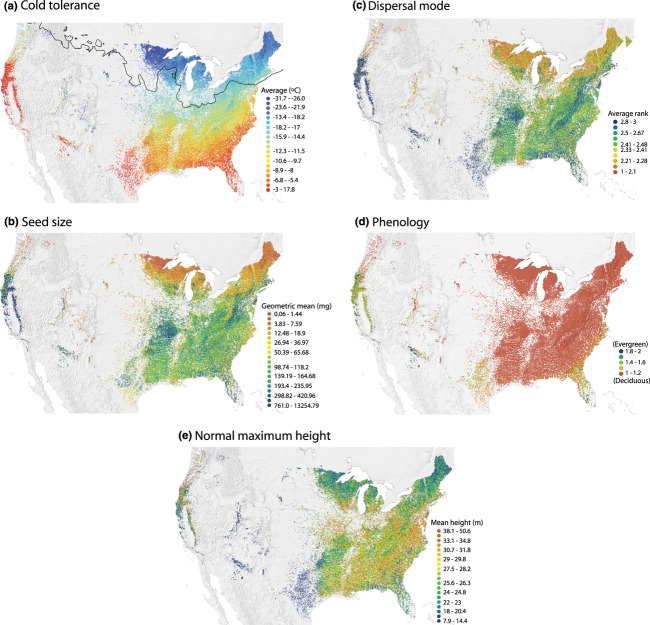
Geographical patterns of (a) mean realized cold tolerance, (b) geometric mean seed size, (c) mean ranked dispersal mode, (d) mean categorized leaf phenology and (e) mean normal maximum height across 91,340 plots for North American angiosperm tree species. The black line in (a) delimits the extent of the ice sheets during the Last Glacial Maximum.

All five traits also contain broad-and local-scale patterning (Fig. 4), but correlations among them range from non-existent (*r *= −0.029 for mean dispersal mode versus mean height) to moderate (*r *= −0.598 for mean dispersal mode versus mean cold tolerance). All patterns are broadly consistent with what we might expect, such as more cold-tolerant species in the north (Fig. 4a), wind-dispersed and small-seeded species composing northern forests (Fig. 4b,c), evergreen-dominated forests to the south (recalling that gymnosperms have been excluded) (Fig. 4d), and shorter trees in arid and semi-arid areas (Fig. 4e). Three traits, mean cold tolerance, mean seed size and mean dispersal mode, also show visibly one of the smaller scale patterns seen in MFA, with sites close to the lower Mississippi River being more similar to sites upriver than to adjacent southern areas (cf. Figs 3 & 4a–c).

**Figure 5:**
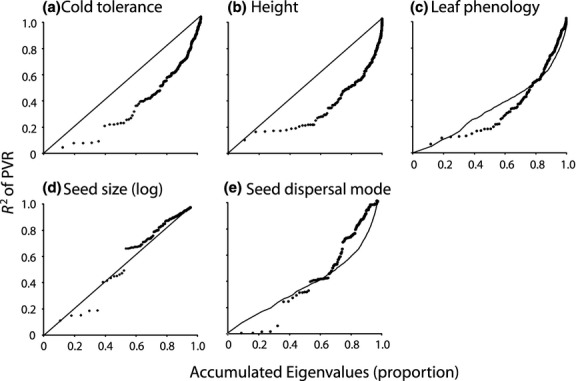
Phylogenetic signal representation curves (black dots) for (a) cold tolerance, (b) height, (c) leaf phenology, (d) seed size and (e) seed dispersal mode of North American tree species. The diagonal in the continuous traits (a,b,d) is the relationship expected under a Brownian motion model of evolution. The Brownian expectations for the discrete traits (c,e) are average *R*^2^ values derived from 250 simulations.

Of the five traits, cold tolerance and height clearly fit an OU model of evolution better than Brownian motion (Table 1), and no trait fits a white noise model (*P *<* *0.001 in all cases). The PSR curves for both cold tolerance and height are also consistent with a niche conservatism interpretation of trait evolution under an OU process (Fig. 5). Seed size displays only small deviations from a Brownian pattern over much of the phylogeny according to PSR curve, with constrained evolution only in the first few eigenvectors, which express variation encompassing the deepest nodes of the phylogeny. For the two discrete traits, the PSR is not statistically different from Brownian motion for seed dispersal mode (*P *=* *0.430) or leaf phenology (*P *=* *0.076), and a similar result was found for the pGLS likelihood test, although the simulation approach to testing PSR for these discrete traits may be more conservative (i.e. both are based on simulating the expected distribution under Brownian motion traits converted into discrete states). However, judged by AIC the OU model is always the best-fit model, regardless of more conservative statistical significance of PSR and pGLS for discrete traits. Irrespective of these differences in results, cold tolerance of trees currently inhabiting North America, as estimated by our realized tolerance measure, has evolved in a manner consistent with niche conservatism, whether defined by Brownian motion or by constrained evolution (Fig. 5a). The same is true for height (Fig. 5b) and perhaps leaf phenology (Fig. 5c), both of which are likely to also represent adaptive traits with respect to tree survival when exposed to freezing temperatures.

**Table 1:** Comparative fits of five traits to alternative evolutionary models for North American angiosperm tree species, using phylogenetic generalized least squares. 2^*^LR_OU–BM_ is twice the likelihood difference comparing the fit of an Ornstein–Uhlenbeck (OU) model against a Brownian motion (BM) model, and 2^*^LR_OU–WN_ compares the fit of an OU model against a white noise (WN) model. *n* represents the number of species with data for each trait. For each analysis the phylogeny was adjusted to include only those species with trait data, and *P*-values for the discrete traits were established by simulating Brownian evolution on the phylogeny and obtaining an empirical distribution for the differences. Akaike weights (*w*AIC), also used to evaluate model fit, are based on exp(−0.5 × ΔAIC) and express the probability that each model is the best among those compared

Trait	*n*	2^*^LR_OU–BM_	*P*	2^*^LR_OU–WN_	*P*	*w*AIC		
						BM	OU	WN
Cold tolerance	500	115.2	< 0.001	205.5	< 0.001	0	1	0
Height	500	362.5	< 0.001	45.4	< 0.001	0	1	0
Leaf phenology	500	64.3	0.376	199.7	< 0.001	0	1	0
Seed size (log)	321	1.8	0.179	443.9	< 0.001	0.12	0.88	0
Seed dispersal mode	479	2.1	0.976	367.8	< 0.001	0.01	0.99	0

The random forest model that included only traits as an explanation of MFA identified realized cold tolerance as the most important trait associated with mean family age across the USA (Table 2). No other trait represented a strong competitor. The model accounted for two-thirds of the variation in MFA, but the risk estimate indicates that there remains substantial variation not accounted for by the traits at our disposal (or perhaps by any traits). The linear regression of MFA against mean cold tolerance accounted for 45.6% of the variance in age. Inclusion of the other four traits in a multiple regression increased the explanatory power of the model to 51.2%. The relatively low coefficient of determination indicates that linear modelling is not able to describe the relationship between traits and MFA as well as the random forest model, but also confirms that most of the variance in mean age explained statistically by traits is captured by mean cold tolerance.

**Table 2:** Variable importance values from random forest models for mean family age of 212 North American angiosperm tree species in 91,340 plots, with the inclusion of trait, broad-scale environmental or local environmental variables in the models. The test risk estimate (squared error rate) of each model is also provided

Predictors	Importance	Risk
Traits		34.6
Cold tolerance	1.000	
Seed size (log)	0.557	
Leaf phenology	0.351	
Dispersal type	0.342	
Height	0.287	
Broad-scale environment		37.1
Minimum temperature (BIO6)	1.000	
Maximum temperature (BIO5)	0.707	
Annual precipitation (BIO12)	0.700	
Summer precipitation (BIO18)	0.699	
Glaciated at Last Glacial Maximum	0.669	
Precipitation in the driest quarter (BIO17)	0.581	
Mean soil moisture	0.218	
Local environment		69.0
Elevation	1.000	
Physiography	0.098	
Slope	0.037	
Aspect	0.025	

Minimum temperature in the coldest month was the most important predictor in the broad-scale environmental random forest model, as predicted (Table 2). None of the other variables competed strongly with minimum temperature, and remotely-sensed soil moisture was a particularly poor predictor. The risk estimate was slightly higher than when using traits as predictors (Table 2) and is large enough to suggest either that we are missing one or more important environmental predictors or that the unexplained variation is stochastic or not under direct environmental control. The linear regression of MFA against minimum temperature accounted for 45.2% of the variance in age (virtually the same as mean cold tolerance), but adding the other broad-scale predictors to a general linear model improved the explanatory power of the environmental variables by only 4.7%, indicating little independent power beyond that provided by minimum temperatures.

The local-environment random forest model fitted the data very poorly. Of the four variables, only elevation had any predictive power (Table 2), and this geographical variable must contain an embedded temperature signal. Thus, across the USA the local variables we were able to generate have minimal or no relationships with mean family age. In a post-hoc analysis we examined small-scale topographical heterogeneity as a potential predictor of localized variation in MFA by calculating the standard deviation in elevations of sites found within each of 1238 drainage basins containing at least 10 FIA sites (drainage map available as a shape file at http://water.usgs.gov/GIS/metadata/usgswrd/XML/ds573_wbdhuc8.xml#stdorder), against which we regressed the standard deviation in MFA. However, elevational heterogeneity of sites within watersheds statistically explained only 1.8% of the variance in heterogeneity in MFA, reinforcing our finding that localized variation in forest phylogenetic structure is difficult to explain using local and semi-local environmental predictors.

**Figure 6:**
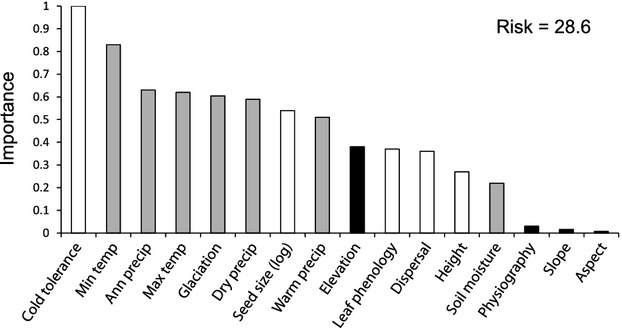
Variable importance values from a random forest model (based on 100 regression trees) of mean family age of North American angiosperm tree species across 91,340 plots, with the inclusion of all trait and environmental predictors in the models. Traits are in white, broad-scale environmental variables are in grey, and local-scale environmental variables are in black. Non-abbreviated variable names are given in Table 2.

The combined random forest model that included all predictors was consistent with the partial models, ranking cold tolerance as the most important predictor, with minimum temperature ranking second (Fig. 6). The risk estimate of the combined model was slightly lower than the separate trait and environment models but still suggests that we are either missing important predictors or that the data contain substantial stochastic variation. The general linear model containing all trait and broad-scale predictors accounted for 56.1% of the variance in age, indicating that linear models cannot capture the complex association between MFA and the predictors as well as the random forest can.

**Figure 7:**
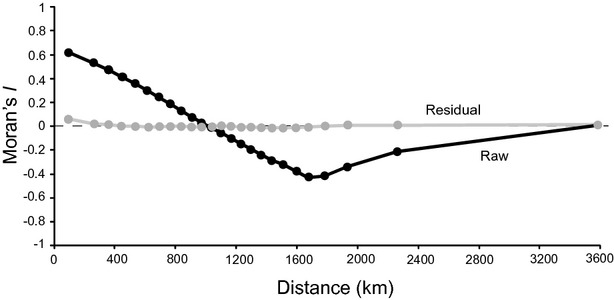
Spatial correlogram of mean family age for North American angiosperm tree species using a random sample of 15,000 sites (raw data and residuals from a random forest model that included five tree traits and eleven environmental variables operating over local or broad scales). All Moran's *I* values for the residuals are between −0.020 and 0.036.

The pattern of spatial structure in the raw data indicates a cline from small to intermediate distances (Fig. 7), reflecting the latitudinal gradient in MFA. There was almost no autocorrelation in the longest distance class because eastern and western forests have very similar age patterns. The residual autocorrelation from the combined random forest model retained almost no detectable spatial autocorrelation at any scale, indicating that the model captured virtually all spatial pattern in MFA and that unexplained variation must reflect a combination of abiotic and/or biotic influences operating over very short distances, stochasticity and sampling error.

## Discussion

All of the patterns predicted by an explanation of the phylogenetic structure of arborescent angiosperms based on phylogenetic niche conservatism of cold tolerance were found in our analyses. First, the angiosperm component of local forest sites across the southern USA is composed of species from older families than are sites further north. Second, of the tree traits available to us, cold tolerance is by far the trait most strongly associated with mean family ages across the USA. Third, minimum temperature represents the environmental variable most strongly associated with mean family age, strongly implicating freezing as the primary climatic driver of patterns of forest taxonomic composition. Fourth, realized cold tolerance, to the extent that it represents a realization of physiological tolerance, has a phylogenetic signal consistent with niche conservatism. The available evidence is thus consistent with a historical interpretation of the structure of forests in the Northern Hemisphere as developed by Latham & Ricklefs (1993), Ricklefs (2005) and Donoghue (2008). Indeed, the current composition of North American forests remains similar to that of *c*. 15 Ma, driven by the mid-Tertiary cooling of the global climate, as described by Graham (1999, p. 233):

Thus, by the end of the early Miocene the older tropical dry forest and much of the notophyllous broad-leaved evergreen vegetation has disappeared. The principal plant communities of North America were the remaining elements of a tropical community along the southern coasts, deciduous forest (sand pine scrub and other components of the pine woods association; oak–chestnut, oak–hickory; southern mixed hardwoods; flood-plain forest), elements of an Appalachian montane coniferous forest, lake state forest (to the far north), shrubland/chaparral–woodland–savanna, mixed hardwood–conifer forest, and western montane coniferous forest.

Although the details of the spatial pattern of mean family age are complex, with much apparent local variation, the explanation for the continental-scale pattern seems *relatively* simple. Minimum winter temperatures are able to account for almost all of the broad-scale and much of the small-scale spatial pattern found in over 90,000 forest sites. We cannot conclude that winter temperature is the only factor determining why trees in the southern half of the USA are, on average, from older families than those in the northern half, but it must play a strong role. We also have not analysed gymnosperms, which might be expected to be at least as strongly associated with rainfall patterns as by temperature gradients. Modern gymnosperms began their initial diversification in the Permian, which was characterized by extensive aridity, and consequently they have a number of traits that provide physiological drought tolerance, permitting them to survive on frozen soils, deep sand and steep slopes (Graham, 1999). The phylogenetic structure of forests dominated by gymnosperms may be substantially different from those containing angiosperms.

Our combined random forest model was unable to account for just over one-quarter of the variation in mean family age, with virtually all of the residual variation being aspatial. Presumably, some of this is due to locally acting environmental factors, but none of the variables we were able to generate that contained small-scale variation could account for more than trivial amounts of local age structure. We also lack any direct measures of local biotic interactions. On the other hand, some of the unexplained variation may be stochasticity or sampling and measurement error. Random dispersal events will not influence broad-scale patterns but may be important over distances of a few kilometres and could generate small-scale variation in community composition. Another obvious source of local variation is non-exhaustive sampling. The FIA protocol consists of sampling four circular plots of 7.3-m radius each (http://fia.fs.fed.us/library/database-documentation/, version 5.1 for Phase 2, p. 10), and any 0.07-h sample will necessarily sample rare tree species imperfectly. That the sampling protocol does miss species is clear from the fact that the FIA database contains records for *c*. 215 currently recognized native angiosperms when well over 400 are known to occur in the contiguous USA (the exact number depends on the definition of trees used). Although we cannot quantify the level of sampling error across the USA, we expect some (perhaps most) of the unexplained variation in mean family age to be due to random or semi-random local dispersal and non-spatially structured sampling error.

An important issue when evaluating community phylogenetic structure as we do here is the use of a higher taxonomic rank to fix ages. What groups of species are combined to compose a family is constantly under revision, and taxonomic decisions will influence clade ages. But although such decisions are not fully objective, they are by no means random. When using a phylogenetic classification system as implemented in APG III (Stevens, 2001), earlier-diverging families are more likely to retain plesiomorphic attributes than later-diverging families. In a biogeographical context the phylogenetic framework can lead to an upscaled version of Hennig's progression rule (Hennig, 1966), in which clades with a series of increasingly derived synapomorphic characters will be found moving away from the group's centre of origin, where clades with plesiomorphic characters dominate (Ashlock, 1974). Given the geological history of angiosperm trees (Latham & Ricklefs, 1993), and the fact that strong spatial structure in the apparent ages of families arises when using either coarse-grained family-level (Hawkins *et al*., 2011) or fine-grained species-level data (this study), it is highly likely that the current angiosperm classification system contains meaningful evolutionary signal at the family rank. Similarly, it is likely that the apomorphies generating the progression rule for tree families moving away from the humid tropics are linked to adaptations for survival in cold and/or dry environments. Finally, it is important to note that our focus on the family rank is not peculiar to this study; Latham & Ricklefs (1993) explicitly claimed that older angiosperm families were strongly impacted by mid-Tertiary cooling, a pattern generally confirmed by our analysis.

**Figure 8:**
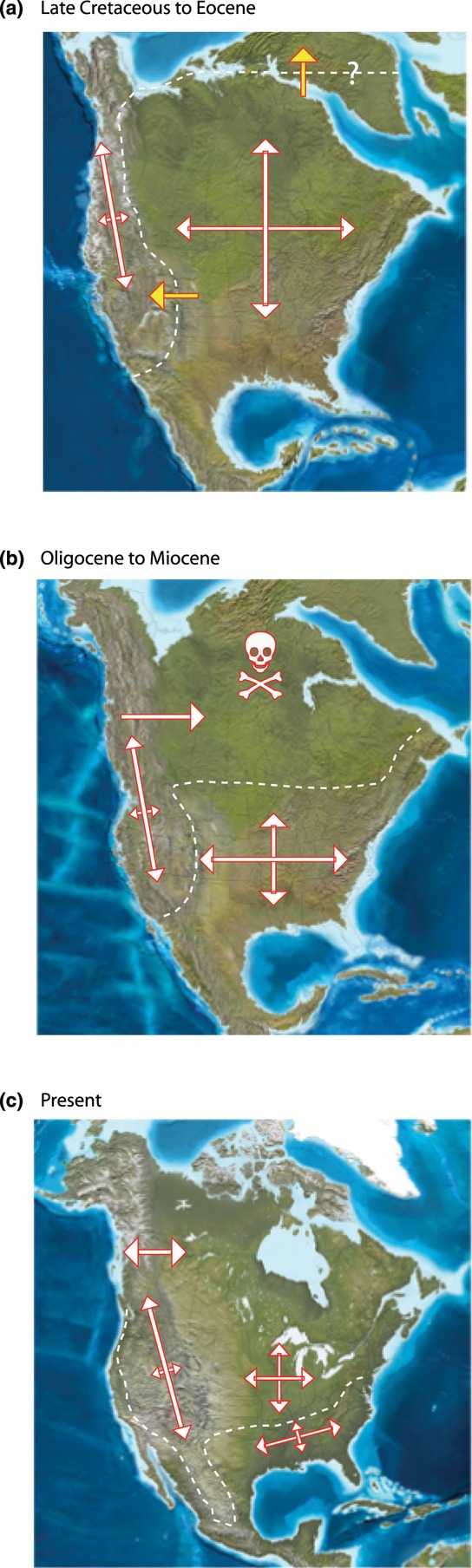
Key features of a hypothesized biogeographical history of angiosperm trees over the last 75 million years in North America. (a) At 60 Ma, during the Laramide orogeny that formed the initial rise of the Rocky Mountains. (b) During mid-Tertiary cooling that started 34 Ma. (c) At the present day (with the continent recently joined to South America). Dashed lines stylistically delimit zones within which freezing due to altitudinal or latitudinal gradients in temperature developed. White arrows represent the addition of species by speciation and dispersal, with longer arrows indicating more such addition. Note the increasing latitudinal restriction outside the zone of freezing through time. Yellow arrows reflect selection gradients to which some tree clades were able to respond via the evolution of cold tolerance. Species are assumed to have been going extinct continuously, but particularly high extinction occurred in cold areas as the freeze line moved south; families comprising species that have been unable to evolve cold tolerance have disappeared from these parts of the continent. The figure is intended to represent spatially the tropical conservatism hypothesis as set out by Wiens & Donoghue (2004), the effects on trees of mid-Tertiary climate change as envisaged by Latham & Ricklefs (1993), and the role of high mountain chains and cool climates at high palaeolatitudes in accounting for the apparent evolution of cold tolerance in some clades prior to the Oligocene. The base maps were generated by Ron Blakey and Colorado Plateau Geosystems and are available at http://www.cpgeosystems.com/index.html (accessed in November, 2012).

Although using family ranks to date the origin of clades generates a spatial pattern consistent with tropical conservatism, there is one key issue that requires potential revision with respect to the scenario of Latham & Ricklefs (1993). The environmental stimulus for climatic niche evolution of trees is presumed to be the mid-Tertiary climate shift initiated at the end of the Eocene (33.9 Ma) and extending into the present. However, of the 35 families for which we were able to obtain fossil-based age estimates (Appendix S3), none can unambiguously be assigned a post-Eocene origin (as also noted and discussed by Latham & Ricklefs, 1993). Thus, there is apparently a substantial temporal mismatch between the timing of the selective event and the ages of the clades that presumably responded. We are left with an important question: if essentially all tree families originated before the Oligocene climate shift, why did taxa in relatively young groups evolve cold tolerance, permitting them to occupy the newly created cold regions, whereas taxa in relatively older families did not, retreating towards the tropics? We have no direct evidence that might account for this, but the initial rise of the Rocky Mountains spanning the Late Cretaceous to the Palaeocene (80–55 Ma; English & Johnston, 2004) may have provided the selective force for the evolution of cold tolerance, perhaps coupled with the development of relatively cold climates at high palaeolatitudes in the Late Cretaceous (Wing & Sues, 1992). That is, the selection for cold tolerance could have begun when angiosperm families were originating well before the Eocene Thermal Optimum, pre-adapting many clades for expansion into the newly created lowland cold climatic regimes generated in the Oligocene (see Fig. 8 for how this hypothesis can account for current patterns in the face of strong niche conservatism). This aspect of the tropical conservatism hypothesis vis-à-vis trees requires additional work, but it does not alter the observation that forest communities to the north are dominated by species from younger families than are those to the south, a pattern that begs for an explanation.

Patterns of trait evolution further point towards phylogenetic niche conservatism as influencing North American tree distributions. Under the interpretations of Losos (2008) and Wiens *et al*. (2010), finding that a trait fits an OU model is consistent with PNC. The pattern of nonlinear evolution slower than BM is also expected under the phylogenetic inertia and niche filling models of Cooper *et al*. (2010). The relationship between PNC and phylogenetic signal is not direct and is not easy to establish in practice, because strong PNC (for instance, very strong stabilizing selection modelled as an OU process with a high α) can eliminate phylogenetic patterns of covariance among species, mimicking a white noise model. There is also no consensus as to whether phylogenetic signal per se (e.g. Brownian motion evolution) is sufficient to be considered phylogenetic niche conservatism, or more constrained patterns of evolution are required. Irrespective, it remains that for the key trait in our analysis, cold tolerance, a substantially better fit to an OU model and the nonlinear PSR curve are evidence for PNC, however defined, because phylogenetic signal exists and the covariance structure among species has been constrained and divergence has been substantially slowed when compared with Brownian expectations. Among the continuous traits seed size comes closest to fitting a Brownian motion model, but it is not strongly associated with mean family age. The discrete traits are also perhaps not conserved as the process is defined by Losos (2008) (although both contain phylogenetic signal), but they are not strong predictors of mean family age.

All average trait values of local tree communities are spatially structured, but average cold tolerance is by far most closely associated with the family-rank ages of the trees. That tree traits are associated with climate has been established in the eastern United States (Swenson & Weiser, 2010), and seed size in particular decreases latitudinally and is known to contain phylogenetic signal (Moles *et al*., 2007). However, Moles *et al*. (2007) attributed the gradient primarily to geographical shifts in plant growth form, which cannot account for the gradient in our data, because all species are trees. Swenson *et al*. (2012) also found geographical patterns in six traits of woody plants across the Western Hemisphere, including maximum height and seed mass, but our data are restricted to angiosperm trees and perhaps for this reason generate different patterns than they found. Although even a cursory examination of the broad-scale patterns of traits invites speculation about adaptive responses of trees to climate, we are focused on understanding why southern forests contain species from older families than northern forests, and only average cold tolerance seems to offer a reasonable explanation.

One smaller-scale pattern across multiple traits merits special mention. Forests from eastern Texas to northern Florida are generally composed of angiosperms that are from older families (Fig. 3), cold intolerant (Fig. 4a), seed-dispersed by animals or unassisted (Fig. 4c), and a mix of deciduous and evergreen species (Fig. 4d). In contrast, forests growing along the lower Mississippi River have averages much more similar to forests in the central and upper mid-western states. This suggests some interesting scenarios. First, as the Laurentide Ice Sheet retreated with the arrival of the current interglacial period, angiosperms colonizing newly exposed sites were primarily cold tolerant, wind dispersed and deciduous. After the drainage of glacial meltwater was re-established to the Gulf of Mexico via the Mississippi River Basin, after having been diverted to the North Atlantic through the Great Lakes and the Gulf of St Lawrence during the Younger Dryas (Broecker *et al*., 1989), significant down-river dispersal from the establishing northern forests probably occurred (see also Graham, 1999, for discussion of this phenomenon), reversing the generalized directional pattern of post-Pleistocene recolonization of northern North America by trees (Davis *et al*., 1986; Ordonez & Williams, 2013). An alternative, that the lower Mississippi retains species compositions that were forced into a refugium as the most recent Ice Age developed, is possible but less likely given the probable distribution of forest refugia in eastern North America (Loehle, 2007). A third possibility is that the regular historical disturbance from Mississippi flooding favoured generalist species that are more similar to those found much further north (Jocque *et al*., 2010). Whichever scenario best describes the patterns along the lower Mississippi River, it illustrates that although cold tolerance is probably a primary driver of local community structure of North American forests, it is not the only one, and explanations for local patterns depend on the region being evaluated.

A final note related to the Laurentide Ice Sheet is that although it necessitated the regeneration of forests *de novo* as it retreated northwards, it seems to have left little imprint as quantified in the environmental model of mean family age. We cannot conclude that its influence was nil, but covariation with the current climatic gradient makes it difficult to identify clear impacts at the continental extent.

Our combined random forest model accounted for almost three-quarters of the variance in mean family age, with almost no residual spatial structure in the data at any scale. This indicates that no additional spatially structured predictors are needed to account for the spatial pattern. But it also leaves open the question of the extent to which the unexplained phylogenetic age structure of angiosperm forest communities represents deterministic, local abiotic/biotic processes or stochasticity arising from random (or neutral) local assembly and/or sampling error. The level of variance accounted for by the model suggests that local processes play a lesser role in community assembly than biogeographical processes, but without direct evidence this remains a supposition. Teasing apart the contributions of local versus regional processes to local community structure remains a core goal of community ecology. Here we have focused primarily on regional factors, which is one step towards understanding community phylogenetic structure at all scales.

## Biosketch

**Bradford A. Hawkins** is interested in ecological and phylogenetic patterns across a range of spatial scales, with a focus on linking local and biogeographical processes.

Author contributions: B.A.H. conceived the idea, created the phylogeny, conducted the geographical analyses and wrote the first draft of the manuscript. M.R. generated the community data and some trait data and conducted parts of the phylogenetic analyses. T.F.R. developed randomization procedures for the phylogenetic analyses, R.F. generated most of the trait data, and J.A.F.D.-F. conducted most of the phylogenetic analyses. All co-authors contributed to the writing of the manuscript.

## SUPPORTING INFORMATION

Appendix S1 Sources used to construct the generic and species nodes of the North American tree phylogeny and the complete phylogeny in Newick format.

Appendix S2 High resolution graphical version of the phylogeny of North American trees.

Appendix S3 List of tree species, including family ages and trait values.
