# Supplementary material for: Community phylogenetics at the biogeographical scale: cold tolerance, niche conservatism and the structure of North American forests
Source: J Biogeogr. 2013 Jul 31;41(1):23–38. doi: 10.1111/jbi.12171 (PMC3920643; doi:10.1111/jbi.12171)
Supplement: Appendix S1 — Sources used to construct the generic and species nodes of the North American tree phylogeny and the complete phylogeny in Newick format. [file jbi0041-0023-sd1.docx]

*Journal of Biogeography*

**SUPPORTING INFORMATION**

**Community phylogenetics at the biogeographical scale: cold tolerance, niche conservatism and the structure of North American forests**

Bradford A. Hawkins, Marta Rueda, Thiago F. Rangel, Richard Field and José Alexandre F. Diniz-Filho

**Appendix S1:** Sources for constructing the generic and species levels of the North American tree phylogeny, followed by a Newick version of the phylogeny.

Aradhya, M. K., Potter, D., Gao, F. & Simon, C. J. (2007) Molecular phylogeny of *Juglans* (Juglandaceae): a biogeographic perspective. *Tree Genetics and Genomes*, **3**, 363–378.

Baker, W. J., Savolainen, V., Asmussen-Lange, C. B., Chase, M. W., Dransfield, J., Forest, F., Harley, M. M., Uhl, N. W. & Wilkinson, M. (2009) Complete generic-level phylogenetic analyses of palms (Arecaceae) with comparisons of supertree and supermatrix approaches. *Systematic Biology*, **58**, 240–256.

Becerra, J. X. & Venable, D. L. (1999) Nuclear ribosomal DNA phylogeny and its implications for evolutionary trends in Mexican *Bursera* (Burseraceae). *American Journal of Botany*, **86**, 1047–1057.

Bortiri, E., Oh, S.-H., Jiang, J., Baggett, S., Granger, A., Weeks, C., Buckingham, M., Potter, D. & Parfitt, D. E. (2001) Phylogeny and systematics of *Prunus* (Rosaceae) as determined by sequence analysis of ITS and the chloroplast *trnL*–*trnF* spacer DNA. *Systematic Botany*, **26**, 797–807.

[Bortiri](http://www.springerlink.com/content/?Author=E.+Bortiri), E., [Vanden Heuvel](http://www.springerlink.com/content/?Author=B.+Vanden+Heuvel), B. & [Potter](http://www.springerlink.com/content/?Author=D.+Potter), D. *(2*006) [Phylogenetic analysis of morphology in *Prunus* reveals extensive homoplasy](http://www.springerlink.com/content/k4w214302j3j8284/). *Plant Systematics and Evolution*, **259**, 53–71.

Bousquet, J., Strauss, S. H. & Li, P. (1992) Complete congruence between morphological and *rbc*L-based molecular phylogenies in birches and related species (Betulaceae). *Molecular Phylogenetics and Evolution*, **9**, 1076–1088.

Buerki, S., Lowry, P. P, II, Alvarez, N., Razafimandimbison, S. G., Küpfer, P. & Callmander M. W. (2010) Phylogeny and circumscription of Sapindaceae revisited: molecular sequence data, morphology and biogeography support recognition of a new family, Xanthoceraceae. *Plant Ecology and Evolution*, **143**, 148–159.

Campbell, C. S., Wojciechowski, M. F., Baldwin, T B. G., Alice, L. A. & Donoghue, M. J. (1997) Persistent nuclear ribosomal DNA sequence polymorphism in the *Amelanchier* agamic complex (Rosaceae). *Molecular Biology and Evolution*, **14**, 81–90.

Campbell, C. S., Evans, R. C., Morgan, D. R., Dickinson, T. A. & Arsenault, M. P. (2007) Phylogeny of subtribe Pyrinae (formerly the Maloideae, Rosaceae): limited resolution of a complex evolutionary history. *Plant Systematics and Evolution*, **266**, 119–145.

Chanderbali, A. S., van der Werff, H. & Renner, S. S. (2001) Phylogeny and historical biogeography of Lauraceae: evidence from the chloroplast and nuclear genomes. *Annals of the Missouri Botanical Garden*, **88**, 104–134.

[Chen Z. D](http://www.ncbi.nlm.nih.gov/pubmed?term=Chen%20ZD%5BAuthor%5D&cauthor=true&cauthor_uid=10449397)., Manchester, S. R. & Sun, H. Y. (1999) Phylogeny and evolution of the Betulaceae as inferred from DNA sequences, morphology, and paleobotany. *American Journal of Botany*, **86**, 1168–1181.

Chen, Z. & Li, J. (2004) Phylogenetics and biogeography of *Alnus* (Betulaceae) inferred from sequences of nuclear ribosomal DNA ITS region. *International Journal of Plant Sciences*, **165**, 325–335.

Clayton, J. W., Fernando, E. S., Soltis, P. S. & Soltis, D. E. (2007) Molecular phylogeny of the tree‐of‐heaven family (Simaroubaceae) based on chloroplast and nuclear markers. *International Journal of Plant Sciences*, **168**, 1325–1339.

[Grimm](http://www.ncbi.nlm.nih.gov/sites/entrez?cmd=search&db=PubMed&term=%20Grimm%20GW%5Bauth%5D), G. W., [Renner](http://www.ncbi.nlm.nih.gov/sites/entrez?cmd=search&db=PubMed&term=%20Renner%20SS%5Bauth%5D), S. S., [Stamatakis](http://www.ncbi.nlm.nih.gov/sites/entrez?cmd=search&db=PubMed&term=%20Stamatakis%20A%5Bauth%5D), A. & [Hemleben](http://www.ncbi.nlm.nih.gov/sites/entrez?cmd=search&db=PubMed&term=%20Hemleben%20V%5Bauth%5D), V. (2006) A nuclear ribosomal DNA phylogeny of *Acer* inferred with maximum likelihood, splits graphs, and motif analysis of 606 Sequences. *Evolutionary Bioinformatics Online*, **2**, 7–22.

[Grimm](http://aob.oxfordjournals.org/search?author1=Guido+W.+Grimm&sortspec=date&submit=Submit), G. W. & [Denk](http://aob.oxfordjournals.org/search?author1=Thomas+Denk&sortspec=date&submit=Submit), T. (2008) ITS Evolution in *Platanus* (Platanaceae): homoeologues, pseudogenes and ancient hybridization. *Annals of Botany*, **101**, 403–419.

Fritsch, P. W., Morton, C. M., Chen, T. & Meldrum, C. (2001) Phylogeny and biogeography of the Styracaceae. *International Journal of Plant Sciences*, **162**, S95–S116.

[Järvinen](http://www.amjbot.org/search?author1=Pia+J%C3%A4rvinen&sortspec=date&submit=Submit), P., [Palmé](http://www.amjbot.org/search?author1=Anna+Palm%C3%A9&sortspec=date&submit=Submit), A., [Morales](http://www.amjbot.org/search?author1=Luis+Orlando+Morales&sortspec=date&submit=Submit), L. O., [Lännenpää](http://www.amjbot.org/search?author1=Mika+L%C3%A4nnenp%C3%A4%C3%A4&sortspec=date&submit=Submit), M., [Keinänen](http://www.amjbot.org/search?author1=Markku+Kein%C3%A4nen&sortspec=date&submit=Submit), M., [Sopanen](http://www.amjbot.org/search?author1=Tuomas+Sopanen&sortspec=date&submit=Submit), T. &  [Lascoux](http://www.amjbot.org/search?author1=Martin+Lascoux&sortspec=date&submit=Submit), M. (2004) Phylogenetic relationships of *Betula* species (Betulaceae) based on nuclear *ADH* and chloroplast *matK* sequences. *American Journal of Botany*, **91**, 1834–1845.

Joey S. & Randall, L. S. (2004) Addressing the “hardest puzzle in American pomology:” Phylogeny of *Prunus* sect. *Prunocerasus* (Rosaceae) based on seven noncoding chloroplast DNA regions. *American Journal of Botany*, **91**, 985–996.

[Hamzeh](http://www.amjbot.org/search?author1=Mona+Hamzeh&sortspec=date&submit=Submit), M. & [Dayanandan](http://www.amjbot.org/search?author1=Selvadurai+Dayanandan&sortspec=date&submit=Submit), S. (2004) Phylogeny of *Populus* (Salicaceae) based on nucleotide sequences of chloroplast *trnT–trnF* region and nuclear rDNA. *American Journal of Botany*, **9**, 1398–1408.

Hardig, T. M., Anttila, C. K. & Brunsfeld, S. J. (2010) A phylogenetic analysis of *Salix* (Salicaceae) based on *matK* and ribosomal DNA sequence data. *Journal of Botany*, 2010, Article ID 197696, 12 pages.

Harrington, M. G., Edwards, K. J., Johnson, S. A., Chase, M. W. & Gadek, P. A. (2005) Phylogenetic inference in Sapindaceae sensu lato using plastid *matK* and *rbcL* DNA sequences. *Systematic Botany*, **30**, 366–382.

Harris. A. J., Xiang, Q.-Y. (J.) & Thomas, D. T. (2009) Phylogeny, origin, and biogeographic history of *Aesculus* L. (Sapindales) – an update from combined analysis of DNA sequences, morphology, and fossils. *Taxon*, **1**, 1–19.

[Hiroshi, A](http://www.amjbot.org/search?author1=Hiroshi+Azuma&sortspec=date&submit=Submit)., [García-Franco](http://www.amjbot.org/search?author1=Jos%C3%A9+G.+Garc%C3%ADa-Franco&sortspec=date&submit=Submit), J. G., [Rico-Gray](http://www.amjbot.org/search?author1=Victor+Rico-Gray&sortspec=date&submit=Submit), V. & [Thien](http://www.amjbot.org/search?author1=Leonard+B.+Thien&sortspec=date&submit=Submit), L. B. (2001) Molecular phylogeny of the Magnoliaceae: the biogeography of tropical and temperate disjunctions. *American Journal of Botany*, **88**, 2275–2285.

Holmgren, K. & Oxelman, B. (2004) Generic limits in *Rhamnus* L. s.l. (Rhamnaceae) inferred from nuclear and chloroplast DNA sequence phylogenies. *Taxon*, **53**, 383–390.

Hugueta, V., Gouyb, M., Normanda, P., Zimpferc, J, F. & Fernandeza, M. P. (2005) Molecular phylogeny of Myricaceae: a reexamination of host–symbiont specificity. *Molecular Phylogenetics and Evolution*, **34**, 557–568.

Hummer, K. E. & Janick, J. (2009) Rosaceae: taxonomy, economic importance, genomics. *Genetics and genomics of Rosaceae, plant genetics and genomics: crops and models* (ed. by K. M. Folta and S. E. Gardiner), pp. 1–17. Springer Science+Business Media, NY.

Kron, K. A., Judd, W. S., Stevens, P. F., Crayn, D. M., Anderberg, A. A., Gadek, P. A., Quinn, C. J. & Luteyn, J. L. (2002) Phylogenetic classification of Ericaceae: molecular and morphological evidence. *Botanical Review*, **68**, 335–423.

Lang, P., Dane, F. & Kubisiak, T. L. (2006) [Phylogeny of *Castanea* (Fagaceae) based on chloroplast *trnT*-*L*-*F* sequence data](http://www.springerlink.com/content/ngt55727113932t2/). [*Tree Genetics and Genomes*](http://www.springerlink.com/content/1614-2942/), **2**, 132–139.

Li, J., Shoup, S. & Chen, Z. (2005) Phylogenetics of *Betula* (Betulaceae) inferred from sequences of nuclear ribosomal DNA. *Rhodora*, **107**, 69–86.

Li, J. (2008) Sequences of low-copy nuclear gene support the monophyly of *Ostrya* and paraphyly of *Carpinus* (Betulaceae). *Journal of Systematics and Evolution*, **46**, 333–340.

Lo, E. Y.Y., Stefanović, S., Christensen, K. I. & Dickinson, T. A. (2009) Evidence for genetic association between East Asian and western North American *Crataegus* L. (Rosaceae) and rapid divergence of the eastern North American lineages based on multiple DNA sequences. *Molecular Phylogenetics and Evolution*, **51**, 157–168.

[Manos P. S](http://www.ncbi.nlm.nih.gov/pubmed?term=Manos%20PS%5BAuthor%5D&cauthor=true&cauthor_uid=10413627)., [Doyle,](http://www.ncbi.nlm.nih.gov/pubmed?term=Doyle%20JJ%5BAuthor%5D&cauthor=true&cauthor_uid=10413627)  J. J. & Nixon, K. C. (1999) Phylogeny, biogeography, and processes of molecular differentiation in *Quercus* subgenus *Quercus* (Fagaceae). *Molecular Biology and Evolution*, **12**, 333–349.

Manos, P. S., Zhe-Kun, Z. & Cannon, C. H. (2001) Systematics of Fagaceae: phylogenetic tests of reproductive trait evolution. *International Journal of Plant Science*, **162**, 1361–1379.

Manos, P. S. & Stone, D. E. (2001) Evolution, phylogeny, and systematics of the Juglandaceae. *Annals of the Missouri Botanical Garden*, **88**, 231–269.

Manos, P. S., Soltis, P. S., Soltis, D. E., Manchester S. R., Oh, S.-H., Bell, C. D., Dilcher, D. L. & Stone, D. E. (2007) Phylogeny of extant and fossil Juglandaceae inferred from the integration of molecular and morphological data sets. *Systematic Biology*, **56**, 412–430.

Miller, A. J., Young, D. A. & Wen, J.  (2001) Phylogeny and biogeography of *Rhus* (Anacardiaceae) based on ITS sequence data. *International* *Journal of Plant Sciences*, **162**, 1401–1407.

Navarro, E., Bousquet, J., Moiroud, A., Munive, A., Piou, D. & Normand, P. (2003) Molecular phylogeny of *Alnus* (Betulaceae), inferred from nuclear ribosomal DNA ITS sequences. *Plant and Soil*, **254**, 207–217.

Pellmyr, O., Segraves, K. A., Althoff, D. M., Balcázar-Lara, M. & Leebens-Mack, J. (2007) The phylogeny of yuccas. *Molecular Phylogenetics and Evolution*, **43**, 493–501.

Potter, D., Gao, F., Bortiri, P. E., Oh, S.-H. & Baggett, S. (2002) Phylogenetic relationships in Rosaceae inferred from chloroplast *matK* and *trnL*–*trnF* nucleotide sequence data. *Plant Systematics and Evolution*, **231**, 77–89.

Potter, D., Eriksson, T., Evans, R. C., Oh, S., Smedmark, J. E. E., Morgan, D. R., Kerr, M., Robertson, K. R., Arsenault, M., Dickinson, T. A. & Campbell, C. S. (2007) Phylogeny and classification of Rosaceae. *Plant Systematics and Evolution*, **266**, 5–43.

[Prince](http://www.amjbot.org/search?author1=Linda+M.+Prince&sortspec=date&submit=Submit) L. M. & [Parks](http://www.amjbot.org/search?author1=Clifford+R.+Parks&sortspec=date&submit=Submit), C. R. (2001) Phylogenetic relationships of Theaceae inferred from chloroplast DNA sequence data. *American Journal of Botany,* **88**, 2309–2320.

[Richardson](http://www.amjbot.org/search?author1=James+E.+Richardson&sortspec=date&submit=Submit), J. E., [Fay](http://www.amjbot.org/search?author1=Michael+F.+Fay&sortspec=date&submit=Submit), M. F.,  [Cronk](http://www.amjbot.org/search?author1=Quentin+C.+B.+Cronk&sortspec=date&submit=Submit), Q. C. B., [Bowman](http://www.amjbot.org/search?author1=Diane+Bowman&sortspec=date&submit=Submit), D. & [Chase](http://www.amjbot.org/search?author1=Mark+W.+Chase&sortspec=date&submit=Submit), M. W. (2000) A phylogenetic analysis of Rhamnaceae using *rbcL* and *trnL*-*F* plastid DNA sequences. *American Journal of Botany*, **87**, 1309–1324.

[Robinson](http://www.springerlink.com/content/?Author=J.+P.+Robinson), J. P., [Harris](http://www.springerlink.com/content/?Author=S.+A.+Harris), S. A. & [Juniper](http://www.springerlink.com/content/?Author=B.+E.+Juniper), B. E. (2001) [Taxonomy of the genus *Malus* Mill. (Rosaceae) with emphasis on the cultivated apple, *Malus domestica* Borkh.](http://www.springerlink.com/content/4177yruqcj6ba4pj/) *Plant Systematics and Evolution*, **226**, 35–58.

Sang-Hun, O. & Manos, P. S. (2008) Molecular phylogenetics and cupule evolution in Fagaceae as inferred from nuclear *CRABS CLAW* sequences. *Taxon*, **57**, 434–451.

Sangtae, K., Chong-Wook, P., Young-Dong, K. & Youngbae, S. (2001) Phylogenetic relationships in family Magnoliaceae inferred from *ndhF* sequences. *American Journal of Botany*, **88**, 717–728.

[Shaw](http://www.amjbot.org/search?author1=Joey+Shaw&sortspec=date&submit=Submit), J. & [Small](http://www.amjbot.org/search?author1=Randall+L.+Small&sortspec=date&submit=Submit), R. L. (2004) Addressing the “hardest puzzle in American pomology:” phylogeny of *Prunus* sect. *Prunocerasus* (Rosaceae) based on seven noncoding chloroplast DNA regions. *American Journal of Botany*, **91**, 985–996.

Simmons, M. P., Savolainen, V., Clevinger, C. C., Archer, R. H. & Davis, J. I. (2001) Phylogeny of the Celastraceae inferred from 26S nuclear ribosomal DNA, phytochrome B, *rbcL, atpB,* and morphology. *Molecular Phylogenetics and Evolution*, **19**, 353–366.

[Stanford](http://www.amjbot.org/search?author1=Alice+M.+Stanford&sortspec=date&submit=Submit), A. M.,  [Harden](http://www.amjbot.org/search?author1=Rachel+Harden&sortspec=date&submit=Submit), R. &  [Parks](http://www.amjbot.org/search?author1=Clifford+R.+Parks&sortspec=date&submit=Submit), C. R. (2000) Phylogeny and biogeography of *Juglans* (Juglandaceae) based on *matK* and ITS sequence data. *American Journal of Botany*, **87**, 872–882.

Sulaiman, S. F., Culham, A. & Harborne, J. B. (2003) Molecular phylogeny of Fabaceae based on *rbcL* sequence data: with special emphasis on the tribe Mimoseae (Mimosoideae). *Pacific Journal of Molecular Biology and Biotechnology*, **11**, 9–35.

Swenson, U., Richardson, J. E. & Bartish, I. V. (2008) Multi-gene phylogeny of the pantropical subfamily Chrysophylloideae (Sapotaceae): evidence of generic polyphyly and extensive morphological homoplasy. *Cladistics*, **24**, 1006–1031.

[Ueda](http://www.springerlink.com/content/?Author=Kunihiko+Ueda), K., [Kosuge](http://www.springerlink.com/content/?Author=Keiko+Kosuge), K. & [Tobe](http://www.springerlink.com/content/?Author=Hiroshi+Tobe), H. (1997) [A molecular phylogeny of Celtidaceae and Ulmaceae (Urticales) based on *rbcL* nucleotide sequences](http://www.springerlink.com/content/d833052h22564384/). *Journal of Plant Research*, **110**, 171–178.

Zona, S. (1999). A monograph of *Sabal* (Arecaceae: Coryphoideae). *Aliso*, **12**, 583–666.

Wannan, B. S. (2006) Analysis of generic relationships in Anacardiaceae. *Blumea*, **51**, 165–195.

Wen, J. & Stuessy, T. F. (1993) The phylogeny and biogeography of *Nyssa* (Cornaceae). *Systematic Botany*, **18**, 68–79.

Wen, J., Berggren, S. T., Lee, C.-H., Ickert-Bond, S., Yi, T.-S., Yoo, K.-O., Xie, L., Shaw, J. & Potter, D. (2008) Phylogenetic inferences in *Prunus* (Rosaceae) using chloroplast *ndhF* and nuclear ribosomal ITS sequences. *Journal of Systematics and Evolution*, **46**, 322–332.

Wiegrefe, S. J., Sytsma, K. J. & Guries, R. P. (1994) Phylogeny of elms (*Ulmus*, Ulmaceae): molecular evidence for a sectional classification. *Systematic Botany*, **19**, 590–612.

[Wurdack](http://www.amjbot.org/search?author1=Kenneth+J.+Wurdack&sortspec=date&submit=Submit), K. J., [Hoffmann](http://www.amjbot.org/search?author1=Petra+Hoffmann&sortspec=date&submit=Submit), P. & [Chase](http://www.amjbot.org/search?author1=Mark+W.+Chase&sortspec=date&submit=Submit), M. W. (2005) Molecular phylogenetic analysis of uniovulate Euphorbiaceae (Euphorbiaceae sensu stricto) using plastid *rbcL* and *trnL*–*trnF* DNA sequences. *American Journal of Botany*, **92**, 1397–1420.

Wilson, P. G., O’Brien, M. M., Heslewood, M. M. & Quinn, C. J. (2005) Relationships within Myrtaceae sensu lato based on a *matK* phylogeny. *Plant Systematics and Evolution*, **251**, 3–19.

Xiang, Q.-Y. (J.), Thomas, D. T., Zhang, W., Manchester, S. R. & Murrell, Z. (2006) Species level phylogeny of the genus *Cornus* (Cornaceae) based on molecular and morphological evidence — implications for taxonomy and Tertiary intercontinental migration. *Taxon*, **55**, 9–30.

Zerega, N. J. C., Clement, W. L., Datwyler, S. L. & Weiblen, G. D. (2005) Biogeography and divergence times in the mulberry family (Moraceae). *Molecular Phylogenetics and Evolution*, **37**, 402–416.

((Illicium_floridanum:89.0,Illicium_parviflorum:89.0):94.0,(((((Liriodendron_tulipifera:36.0,((Magnolia_acuminata:18.0,(Magnolia_grandiflora:9.0,Magnolia_virginiana:9.0):9.0):9.0,(Magnolia_macrophylla:18.0,(Magnolia_tripetala:9.0,Magnolia_fraseri:9.0):9.0):9.0):9.0):40.0,(Annona_glabra:25.0,Asimina_triloba:25.0):51.0):23.0,((Persea_borbonia:6.5,Persea_palustris:6.5):6.5,(Sassafras_albidum:9.75,((Licaria_triandra:3.25,Umbellularia_californica:3.25):3.25,Nectandra_coriacea:6.5):3.25):3.25):86.0):23.0,Canella_winterana:122.0):43.0,(((Roystonea_regia:33.0,((Washingtonia_filifera:17.6,(Acoelorrhaphe_wrightii:8.8,Serenoa_repens:8.8):8.8):8.799999,((Sabal_minor:13.2,(Sabal_palmetto:6.6,Sabal_mexicana:6.6):6.6):6.599999,((Leucothrinax_morrisii:6.6,Coccothrinax_argentata:6.6):6.6,Thrinax_radiata:13.2):6.599999):6.6):6.6):62.5,(((Yucca_brevifolia:29.333334,(Yucca_carnerosana:22.0,(Yucca_treculeana:14.666667,(Yucca_rostrata:7.333333,Yucca_elata:7.333333):7.333333):7.333333):7.333334):7.333334,(Yucca_mohavensis:18.333334,Yucca_schottii:18.333334):18.333334):7.333333,Yucca_faxoniana:44.0,Yucca_gloriosa:44.0,Yucca_aloifolia:44.0):51.5):51.5,((Platanus_occidentalis:99.0,Platanus_racemosa:99.0):30.0,(((((((((Fagus_grandifolia:31.0,((Castanea_ozarkensis:19.518518,(Castanea_dentata:9.759259,Castanea_pumila:9.759259):9.759259):9.75926,(Chrysolepis_chrysophylla:27.555555,(Lithocarpus_densiflorus:25.833334,((Quercus_palmeri:12.055555,Quercus_chrysolepis:12.055555):12.055555,((Quercus_arkansana:18.657408,Quercus_coccinea:18.657408,Quercus_ellipsoidalis:18.657408,Quercus_emoryi:18.657408,Quercus_georgiana:18.657408,Quercus_canbyi:18.657408,Quercus_ilicifolia:18.657408,Quercus_imbricaria:18.657408,(((Quercus_palustris:5.597222,Quercus_rubra:5.597222):5.597222,(Quercus_incana:7.462963,(Quercus_kelloggii:3.731482,Quercus_agrifolia:3.731482):3.731482):3.731482):3.731482,((Quercus_myrtifolia:5.597222,Quercus_shumardii:5.597222):5.597222,(Quercus_laevis:7.462963,(Quercus_nigra:3.731482,Quercus_laurifolia:3.731482):3.731482):3.731482):3.731482):3.731482,Quercus_marilandica:18.657408,Quercus_phellos:18.657408,Quercus_hypoleucoides:18.657408,Quercus_velutina:18.657408,(Quercus_falcata:9.328704,Quercus_pagoda:9.328704):9.328704,Quercus_gravesii:18.657408):3.731482,((Quercus_ajoensis:16.533333,((Quercus_muehlenbergii:6.2,Quercus_prinoides:6.2):6.2,((Quercus_mohriana:4.133333,Quercus_pungens:4.133333):4.133333,(Quercus_oblongifolia:4.133333,Quercus_grisea:4.133333):4.133333):4.133333):4.133333):4.133333,Quercus_douglasii:20.666666,Quercus_austrina:20.666666,Quercus_bicolor:20.666666,Quercus_sinuata:20.666666,Quercus_glaucoides:20.666666,Quercus_havardii:20.666666,Quercus_oglethorpensis:20.666666,Quercus_toumeyi:20.666666,(((((((((((Quercus_alba:1.722222,Quercus_macrocarpa:1.722222):1.722222,Quercus_garryana:3.444444):1.722222,Quercus_stellata:5.166667):1.722222,Quercus_michauxii:6.888889):1.722222,(Quercus_lobata:4.305555,Quercus_chapmanii:4.305555):4.305555):1.722222,Quercus_dumosa:10.333333):1.722222,(Quercus_gambelii:6.027778,Quercus_arizona:6.027778):6.027778):1.722222,Quercus_rugosa:13.777778):1.722222,Quercus_engelmannii:15.5):1.722221,(Quercus_wislizeni:8.611111,Quercus_lyrata:8.611111):8.611111):1.722223,Quercus_virginiana:18.944445):1.722221):1.722223):1.722221):1.722223):1.722221):1.722223):1.722222):24.0,(((((Myrica_heterophylla:10.666667,Myrica_pensylvanica:10.666667):10.666667,Myrica_cerifera:21.333334):10.666667,Myrica_californica:32.0,Myrica_inodora:32.0):7.666668,(((Carya_floridana:1.333333,Carya_glabra:1.333333,Carya_laciniosa:1.333333,Carya_myristicaeformis:1.333333,Carya_ovata:1.333333,Carya_pallida:1.333333,Carya_texana:1.333333,Carya_alba:1.333333):1.333333,(Carya_aquatica:1.333333,Carya_cordiformis:1.333333,Carya_illinoensis:1.333333):1.333333):1.333333,(Juglans_cinerea:3.2,(Juglans_major:2.4,((Juglans_californica:0.8,Juglans_hindsii:0.8):0.8,(Juglans_nigra:0.8,Juglans_microcarpa:0.8):0.8):0.8):0.8):0.8):35.666668):7.666664,(((Betula_occidentalis:14.285714,(Betula_populifolia:11.428572,(Betula_papyrifera:8.571428,(Betula_nigra:5.714286,(Betula_alleghaniensis:2.857143,Betula_lenta:2.857143):2.857143):2.857142):2.857143):2.857142):2.857142,((Ostrya_knowltonii:5.714285,Ostrya_virginiana:5.714285):5.714285,Carpinus_caroliniana:11.428571):5.714286):2.857143,(((((Alnus_rubra:3.333333,Alnus_incana:3.333333):3.333333,(Alnus_oblongifolia:3.333333,Alnus_rhombifolia:3.333333):3.333333):3.333333,Alnus_serrulata:10.0):3.333333,Alnus_maritima:13.333333):3.333333,Alnus_viridis:16.666666):3.333333):27.333332):7.666667):42.0,(((Cowania_mexicana:33.0,((Cercocarpus_ledifolius:11.0,Cercocarpus_breviflorus:11.0):11.0,Cercocarpus_betuloides:22.0):11.0):11.0,(((Prunus_emarginata:28.430767,(((Prunus_myrtifolia:7.107692,Laurocerasus_caroliniana:7.107692,Laurocerasus_ilicifolia:7.107692):7.107692,(Prunus_serotina:7.107692,Padus_virginiana:7.107692):7.107692):7.107693,Prunus_pensylvanica:21.323076):7.107691):7.107693,((((((Armeniaca_dasycarpa:5.076923,Prunus_angustifolia:5.076923):5.076923,Prunus_umbellata:10.153846):5.076922,Prunus_alleghaniensis:15.230768):5.076923,(Prunus_lanata:15.230768,((Prunus_hortulana:5.076923,Prunus_munsoniana:5.076923):5.076923,Prunus_mexicana:10.153846):5.076922):5.076923):5.076923,Prunus_subcordata:25.384615):5.076921,Emplectocladus_fremontii:30.461536):5.076923):5.076923,(Vauquelinia_californica:37.23077,(Heteromeles_salicifolia:33.846153,((((Crataegus_saligna:15.794872,(Crataegus_erythropoda:7.897436,Crataegus_douglasii:7.897436):7.897436):7.897436,((Crataegus_columbiana:13.538461,(Crataegus_coccinoides:6.76923,Crataegus_pruinosa:6.76923,Oxyacantha_succulenta:6.76923,Crataegus_intricata:6.76923,Crataegus_crusgalli:6.76923,Mespilus_flava:6.76923,Crataegus_tracyi:6.76923):6.76923):6.769231,(Mespilus_punctata:16.923077,(Crataegus_viridis:13.538462,(((Mespilus_coccinea:3.384615,Oxyacantha_mollis:3.384615):3.384615,Crataegus_flabellata:6.769231):3.384615,(Crataegus_uniflora:5.076923,Crataegus_aestivalis:5.076923):5.076923):3.384616):3.384615):3.384615):3.384617):3.384615,Crataegus_phaenopyrum:27.076923):3.384615,((Amelanchier_sanguinea:11.423077,Amelanchier_arborea:11.423077,Amelanchier_interior:11.423077):11.423077,((Aucuparia_americana:7.615385,Pyrus_decora:7.615385,Sorbus_sitchensis:7.615385):7.615385,(Sorbus_coronaria:7.615385,Malus_fusca:7.615385):7.615385):7.615384):7.615385):3.384615):3.384617):3.384613):3.384615):41.0,((((((((Frangula_betulaefolia:10.333333,Frangula_californica:10.333333):10.333333,Frangula_caroliniana:20.666666):10.333334,Frangula_purshiana:31.0):10.333332,Rhamnus_crocea:41.333332):10.333336,(Krugiodendron_ferreum:34.444447,(Condalia_hookeri:17.222223,Condalia_globosa:17.222223):17.222223):17.222221):10.333333,((Colubrina_arborescens:20.666666,Colubrina_cubensis:20.666666):20.666666,(Ceanothus_spinosus:20.666666,Ceanothus_thyrsiflorus:20.666666):20.666666):20.666666):8.333328,Lepargyrea_argentea:70.333328):8.333336,((Planera_aquatica:66.0,((Ulmus_alata:26.4,Ulmus_americana:26.4):26.4,(Ulmus_thomasii:39.599998,(Ulmus_serotina:26.4,(Ulmus_rubra:13.2,Ulmus_crassifolia:13.2):13.2):13.199999):13.200001):13.2):6.333336,(((Trema_lamarckiana:18.5,Trema_micrantha:18.5):18.5,((Celtis_laevigata:12.333333,Celtis_occidentalis:12.333333):12.333333,Celtis_lindheimeri:24.666666,Celtis_ehrenbergiana:24.666666,Celtis_tenuifolia:24.666666):12.333333):17.666668,(((Ficus_aurea:11.666667,Ficus_citrifolia:11.666667):11.666667,Maclura_pomifera:23.333334):11.666667,(Morus_celtidifolia:17.5,Morus_rubra:17.5):17.5):19.666668):17.666668):6.333328):6.333333):12.0):2.5,((((((((Acacia_farnesiana:9.333333,Acacia_macracantha:9.333333,Acacia_tortuosa:9.333333):9.333333,(Acacia_wrightii:9.333333,Acacia_greggii:9.333333):9.333333):9.333334,((Leucaena_pulverulenta:9.333333,Leucaena_retusa:9.333333,Leucena_leucocephala:9.333333):9.333333,(Prosopis_juliflora:9.333333,Prosopis_pubescens:9.333333):9.333333):9.333334):9.333332,((Ebenopsis_ebano:12.444444,Pithecellobium_keyense:12.444444,Havardia_pallens:12.444444,Pithecellobium_unguiscati:12.444444):12.444444,Lysiloma_latisiliquum:24.888887):12.444445):9.333336,(((Gleditsia_aquatica:11.666667,Gleditsia_triacanthos:11.666667):11.666667,Gymnocladus_dioica:23.333334):11.666666,(Caesalpinia_mexicana:23.333334,(Parkinsonia_aculeata:11.666667,Cercidium_floridum:11.666667,Cercidium_macrum:11.666667,Cercidium_microphyllum:11.666667):11.666667):11.666666):11.666668):9.333332,((Cladrastis_kentukea:24.5,Sophora_affinis:24.5):24.5,((Psorothamnus_spinosus:21.0,Eysenhardtia_polystachya:21.0):21.0,(Sophora_secundiflora:35.0,((Olneya_tesota:21.0,(Robinia_hispida:14.0,Robinia_neomexicana:14.0,(Robinia_pseudoacacia:7.0,Robinia_viscosa:7.0):7.0):7.0):7.0,Piscidia_piscipula:28.0):7.0):7.0,(Erythrina_flabelliformis:21.0,Erythrina_herbacea:21.0):21.0):7.0):7.0):7.0,(Cercis_canadensis:31.5,Cercis_occidentalis:31.5):31.5):20.0,Suriana_maritima:83.0):16.5):2.5,(((Schaefferia_frutescens:42.75,(Canotia_holacantha:28.5,(Euonymus_atropurpureus:14.25,Euonymus_occidentalis:14.25):14.25):14.25):14.25,(Maytenus_phyllanthoides:28.5,Crossopetalum_rhacoma:28.5):28.5):40.0,((((((((((Salix_gooddingii:14.0,Salix_nigra:14.0,Salix_laevigata:14.0,Salix_bonplandiana:14.0,Salix_caroliniana:14.0):14.0,(Salix_amygdaloides:14.0,Salix_floridana:14.0):14.0,Salix_lasiandra:28.0):14.0,((Salix_taxifolia:21.0,(Salix_exigua:10.5,Salix_interior:10.5):10.5,Salix_melanopsis:21.0):10.5,(Salix_pellita:21.0,Salix_arbusculoides:21.0,Salix_petiolaris:21.0,(Salix_rigida:10.5,Salix_eriocephala:10.5):10.5,(Salix_tracyi:10.5,Salix_lasiolepis:10.5):10.5,(Salix_discolor:10.5,Salix_hookeriana:10.5,Salix_scouleriana:10.5):10.5,Salix_sitchensis:21.0,(Salix_pyrifolia:10.5,Salix_monticola:10.5):10.5):10.5):10.5):10.5,(Salix_lucida:35.0,(Salix_alaxensis:17.5,Salix_bebbiana:17.5):17.5):17.5):10.5,(((Populus_tremuloides:16.800001,Populus_tremula:16.800001):16.800001,(Populus_angustifolia:16.800001,Populus_balsamifera:16.800001):16.800001):16.799999,(((Populus_deltoides:12.6,Populus_heterophylla:12.6,Populus_palmeri:12.6):12.6,Populus_fremontii:25.200001):12.599998,Populus_trichocarpa:37.799999):12.600002):12.6):5.800003,Rhizophora_mangle:68.800003):5.799995,Drypetes_lateriflora:74.599998):5.800003,Byrsonima_lucida:80.400002):5.799995,Chrysobalanus_icaco:86.199997):5.8,((Hippomane_mancinella:42.5,Sebastiana_bilocularis:42.5):42.5,Gymnanthes_lucida:85.0):7.0):5.0):5.0):4.5,(Guaiacum_angustifolium:70.0,Guaiacum_sanctum:70.0):36.5):4.5,((((((Tilia_americana:19.5,Tilia_caroliniana:19.5):19.5,(Fremontodendron_californicum:19.5,Fremontodendron_mexicanum:19.5):19.5):34.5,(Capparis_cynophallophora:43.0,Koeberlinia_spinosa:43.0):30.5):30.5,(((((((((Schmaltzia_integrifolia:9.142858,Schmaltzia_ovata:9.142858):9.142858,(Schmaltzia_kearneyi:9.142858,Schmaltzia_choriophylla:9.142858):9.142858):9.142857,((Schmaltzia_copallinum:9.142858,Rhus_lanceolata:9.142858):9.142858,(Rhus_glabra:9.142858,Rhus_typhina:9.142858):9.142858):9.142857):9.142859,Cotinus_obovatus:36.57143):9.142857,Pistacia_mexicana:45.714287):9.142857,Metopium_toxiferum:54.857143):9.142858,(Toxicodendron_vernix:32.0,Malosma_laurina:32.0):32.0):3.5,(Bursera_simaruba:64.0,(Bursera_fagaroides:32.0,Bursera_microphylla:32.0):32.0):3.5):3.5,(Alvaradoa_amorphoides:66.800003,((((Dodonaea_viscosa:15.75,Hypelate_trifoliata:15.75,Exothea_paniculata:15.75):15.75,(Ungnadia_speciosa:21.0,(Sapindus_drummondii:10.5,Sapindus_saponaria:10.5):10.5):10.5):10.5,((((Aesculus_sylvatica:14.7,(Aesculus_pavia:7.35,Aesculus_glabra:7.35):7.35):7.349999,Aesculus_flava:22.049999):7.35,Aesculus_californica:29.4):7.35,((((((Acer_spicatum:5.25,Acer_circinatum:5.25):5.25,Acer_pensylvanicum:10.5):5.25,(Acer_negundo:7.875,Acer_glabrum:7.875):7.875):5.25,Acer_macrophyllum:21.0):5.25,Acer_saccharum:26.25):5.25,(Acer_rubrum:15.75,Acer_saccharinum:15.75):15.75):5.25):5.25):20.599998,(((Castela_emoryi:50.0,(Leitneria_floridana:25.0,Simarouba_amara:25.0):25.0):4.200001,Swietenia_mahagoni:54.200001):4.200001,((Ptelea_baldwinii:20.0,Ptelea_trifoliata:20.0):20.0,(Amyris_balsamifera:20.0,Amyris_elemifera:20.0):20.0,Helietta_parvifolia:40.0,(Zanthoxylum_americanum:20.0,Zanthoxylum_clavaherculis:20.0,Zanthoxylum_spinosum:20.0,Zanthoxylum_fagara:20.0):20.0):18.400002):4.199997):4.200005):4.2):33.0):2.333336,(Staphylea_bolanderi:37.0,Staphylea_trifolia:37.0):69.333336):2.333328,((Conocarpus_erectus:24.0,Laguncularia_racemosa:24.0):65.0,(((Calyptranthes_pallens:29.666666,Calyptranthes_zuzygium:29.666666):29.666666,(((Eugenia_axillaris:14.833333,Eugenia_confusa:14.833333,Eugenia_foetida:14.833333):14.833333,Myrcianthes_fragrans:29.666666):14.833334,Mosiera_longipes:44.5):14.833332):14.833332,Tetrazygia_bicolor:74.166664):14.833333):19.666664):2.333333):5.0,(Hamamelis_virginiana:103.0,Liquidambar_styraciflua:103.0):13.0):5.0,((Schoepfia_schreberi:99.0,Ximenia_americana:99.0):19.666664,(((Coccoloba_diversifolia:42.0,Coccoloba_uvifera:42.0):64.0,(Guapira_discolor:64.0,(Cylindropuntia_fulgida:22.0,(Cereus_thurberi:11.0,Carnegiea_giganteus:11.0):11.0):42.0):42.0):10.333336,(((((Cornus_alternifolia:33.5,((Cornus_foemina:11.166667,Cornus_drummondii:11.166667):11.166667,Cornus_glabrata:22.333334):11.166666):11.166668,Cornus_sericea:44.666668):11.166664,(Cornus_florida:27.916666,Cornus_nuttallii:27.916666):27.916666):11.166667,(Nyssa_aquatica:44.666668,(Nyssa_ogeche:22.333334,Nyssa_sylvatica:22.333334):22.333334):22.333334):47.0,(((((((Sideroxylon_foetidissimum:15.2,Sideroxylon_salicifolium:15.2):15.2,Manilkara_jaimiqui:30.4):15.199999,(Sideroxylon_celastrinum:22.799999,Sideroxilon_lanuginosum:22.799999,Sideroxylon_lycioides:22.799999,Sideroxylon_tenax:22.799999):22.799999):15.200001,Chrysophyllum_oliviforme:60.799999):15.200001,((Diospyros_texana:30.0,Diospyros_virginiana:30.0):30.0,(Jacquinia_keyensis:44.0,(Ardisia_escallonioides:22.0,Myrsine_cubana:22.0):22.0):16.0):16.0):16.0,(((Gordonia_lasianthus:44.333332,(Stewartia_malacodendron:22.166666,Stewartia_ovata:22.166666):22.166666):22.166668,((((Halesia_carolina:12.666667,Carlomohria_parviflora:12.666667):12.666667,Halesia_diptera:25.333334):12.666667,Styrax_grandifolia:38.0):14.25,Symplocos_tinctoria:52.25):14.25):14.25,(Clethra_acuminata:69.5,((Cliftonia_monophylla:47.0,Cyrilla_racemiflora:47.0):11.25,((((Chamaedaphne_latifolia:4.5,Elliottia_racemosa:4.5):4.5,((Hymenanthes_macrophylla:3.0,Hymenanthes_catawbiensis:3.0):3.0,Hymenanthes_maxima:6.0):3.0):3.0,((Batodendron_arboreum:4.0,Xolisma_ferruginea:4.0):4.0,Oxydendrum_arboreum:8.0):4.0):3.0,((Arbutus_texana:5.0,Arbutus_arizonica:5.0):5.0,Arbutus_menziesii:10.0):5.0):43.25):11.25):11.25):11.25):18.666664,((Garrya_elliptica:104.0,(((Casasia_clusiifolia:28.5,Psychotria_nervosa:28.5):28.5,(Pinckneya_bracteata:42.75,(Cephalanthus_occidentalis:28.5,Hamelia_patens:28.5,(Guettarda_elliptica:14.25,Guettarda_scabra:14.25):14.25):14.25):14.25):39.5,(((((Chionanthus_virginicus:30.0,Osmanthus_americanus:30.0,(Forestiera_acuminata:15.0,Forestiera_angustifolia:15.0,Forestiera_phillyreoides:15.0,Forestiera_segregata:15.0):15.0):15.0,((Fraxinus_dipetala:27.5,(Fraxinus_anomala:13.75,Fraxinus_quadrangulata:13.75):13.75):13.75,(Fraxinus_cuspidata:37.5,((Fraxinus_americana:30.0,(Fraxinus_papillosa:26.25,(Fraxinus_latifolia:22.5,(Fraxinus_caroliniana:18.75,(Fraxinus_texensis:15.0,(Fraxinus_pennsylvanica:11.25,(Fraxinus_velutina:7.5,(Fraxinus_berlandieriana:3.75,Fraxinus_profunda:3.75):3.75):3.75):3.75):3.75):3.75):3.75):3.75):3.75,((Fraxinus_greggii:11.25,Fraxinus_gooddingii:11.25):11.25,Fraxinus_nigra:22.5):11.25):3.75):3.75):3.75):29.0,((Avicennia_germinans:30.0,Citharexylum_spinosum:30.0):22.0,(Amphitecna_latifolia:25.0,(Chilopsis_linearis:16.666666,(Catalpa_bignonioides:8.333333,Catalpa_speciosa:8.333333):8.333333):8.333333):27.0):22.0):7.5,((Cordia_boissieri:28.5,Cordia_sebestena:28.5):28.5,(Bourreria_ovata:28.5,Ehretia_anacua:28.5):28.5):24.5):7.5,Solanum_erianthum:89.0):7.5):7.5):3.333336,((Ilex_krugiana:45.0,(Ilex_amelanchier:36.0,((Ilex_cassine:18.0,(Ilex_laevigata:9.0,Ilex_verticillata:9.0):9.0):9.0,Ilex_opaca:27.0):9.0):9.0,((Ilex_decidua:15.0,Ilex_montana:15.0):15.0,Ilex_vomitoria:30.0):15.0,(Ilex_coriacea:22.5,Ilex_ambigua:22.5):22.5):55.0,(Artemisia_tridentata:77.666664,(Aralia_spinosa:55.333332,((((Sambucus_canadensis:8.25,Sambucus_nigra:8.25):8.25,Sambucus_racemosa:16.5):8.25,Sambucus_velutina:24.75):8.25,((((Viburnum_prunifolium:6.6,Viburnum_rufidulum:6.6):6.6,Viburnum_lentago:13.2):6.599999,Viburnum_nudum:19.799999):6.6,Viburnum_tridentatum:26.4):6.6):22.333332):22.333332):22.333334):7.333336):3.333328):3.333333):2.333336):2.333328):2.333333):8.0):18.0):18.0):18.0):143.0;
