# Supplementary figures and images for: Community phylogenetics at the biogeographical scale: cold tolerance, niche conservatism and the structure of North American forests
Source: J Biogeogr. 2013 Jul 31;41(1):23–38. doi: 10.1111/jbi.12171 (PMC3920643; doi:10.1111/jbi.12171)

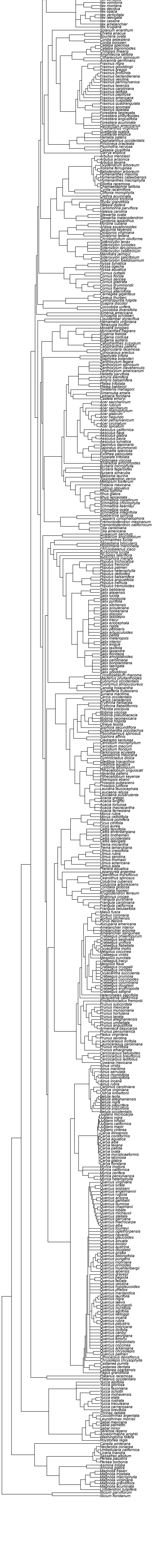

Supplement: Appendix S2 — High resolution graphical version of the phylogeny of North American trees. [file jbi0041-0023-sd2.pdf]
