# Supplementary material for: Community phylogenetics at the biogeographical scale: cold tolerance, niche conservatism and the structure of North American forests
Source: J Biogeogr. 2013 Jul 31;41(1):23–38. doi: 10.1111/jbi.12171 (PMC3920643; doi:10.1111/jbi.12171)
Supplement: Appendix S3 — List of tree species, including family ages and trait values. [file jbi0041-0023-sd3.docx]

*Journal of Biogeography*

**SUPPORTING INFORMATION**

**Community phylogenetics at the biogeographical scale: cold tolerance, niche conservatism and the structure of North American forests**

Bradford A. Hawkins, Marta Rueda, Thiago F. Rangel, Richard Field and José Alexandre F. Diniz-Filho

**Appendix S3:** Species list of North American angiosperm trees, with family ages and trait values. Ages based on Davies *et al*. (2004) are provided for all families in North America. For those families represented in the US Forest Service’s Forest Inventory and Analysis (FIA) database (sampled species are marked with an *), age estimates are also given based on Bell *et al*. (2010) and on fossils provided by the sources listed. The clock-based source closest to each fossil-based estimate is in bold. Fossil-based age estimates were not found for three families; in 11 families the closest clock-based estimate could not be determined with confidence; in the Boraginaceae the taxonomic breadth of the clade was refined by APG III (Angiosperm Phylogeny Group, 2009), so ages are not comparable across studies; and in the remaining cases the ages of Davies *et al*. were closer in 15 and those of Bell *et al*. were closer in 8. But because species were assigned the ages of families for the geographic analysis of mean family age, the 15 vs. 8 difference between the family age estimates of Davies *et al*. and Bell *et al*. translates into 152 vs. 28 species, respectively; that is, the Davies *et al*. age estimates are closer to the fossil-based estimates for 84% of the species in the disputed families for which fossil-based ages are available.

| FAMILY | SPECIES | Family age (Ma) [Davies *et al*.] | Cold Tolerance (degrees C) | Seed Size (mg) | Dispersal Type | Leaf Phenology | Height (m) | Family age (Bell et al.] | Family age [fossil] | Fossil source |
| --- | --- | --- | --- | --- | --- | --- | --- | --- | --- | --- |
| Adoxaceae | *Sambucus canadensis* L. | 86.272 | -12.8 | 2.6 | 2 | 2 | 6.10 |  |  |  |
| Adoxaceae | *Sambucus nigra* L. |  | -20.6 | 2.21 | 2 | 2 | 6.10 |  |  |  |
| Adoxaceae | *Sambucus racemosa* L. |  | -18.5 | 2.13 | 2 | 2 | 14.94 |  |  |  |
| Adoxaceae | *Sambucus velutina* Durand & Hilg. |  | -2.8 |  | 2 | 2 | 10.06 |  |  |  |
| Adoxaceae | *Viburnum lentago* L. |  | -20.4 | 74.16 | 2 | 2 | 10.06 |  |  |  |
| Adoxaceae | *Viburnum nudum* L. |  | -4.1 |  | 2 | 2 | 6.10 |  |  |  |
| Adoxaceae | *Viburnum prunifolium* L. |  | -8.4 | 97.35 | 2 | 2 | 10.06 |  |  |  |
| Adoxaceae | *Viburnum rufidulum* Raf. |  | -3.2 |  | 2 | 2 | 9.14 |  |  |  |
| Adoxaceae | *Viburnum tridentatum* Killip & Smith |  | 7.2 |  | 2 | 2 | 9.14 |  |  |  |
| Altingiaceae | **Liquidambar styraciflua* L. | 101.626 | -3.5 | 5 | 3 | 2 | 40.23 | 99 | 88.5–90.4 | Zhou *et a*l., 2001 |
| Anacardiaceae | **Cotinus obovatus* Raf. | 42.881 | -1.1 | 8.95 | 3 | 2 | 11.89 | **64** | 55–65 | Pell, 1995 |
| Anacardiaceae | *Malosma laurina* (Nutt.) Nutt. ex Abrams |  | 4.7 |  | 2 | 1 | 4.88 |  |  |  |
| Anacardiaceae | **Metopium toxiferum* (L.) Krug & Urb. |  | 17.6 |  | 2 | 1 | 11.89 |  |  |  |
| Anacardiaceae | *Pistacia mexicana* Kunth |  | 9.1 |  | 2 | 2 | 10.06 |  |  |  |
| Anacardiaceae | *Rhus glabra* L. |  | -21.3 | 8.7 | 2 | 2 | 7.01 |  |  |  |
| Anacardiaceae | *Rhus lanceolata* (A. Gray) Britton |  | 2.1 | 7.84 | 2 | 2 | 10.06 |  |  |  |
| Anacardiaceae | *Rhus typhina* L. |  | -16.4 | 11.2 | 2 | 2 | 10.06 |  |  |  |
| Anacardiaceae | *Schmaltzia choriophylla* (Wooton & Standl.) F.A. Barkley |  | 0.1 | 19.5 | 2 | 1 | 4.88 |  |  |  |
| Anacardiaceae | *Schmaltzia copallinum* (L.) Small |  | -10.8 | 8.4 | 2 | 2 | 7.92 |  |  |  |
| Anacardiaceae | *Schmaltzia integrifolia* (Nutt.) F.A. Barkley |  | 3.5 | 54.9 | 2 | 1 | 7.92 |  |  |  |
| Anacardiaceae | *Schmaltzia kearneyi* (F.A. Barkley) F.A. Barkley |  | 8.6 |  | 2 | 1 | 4.88 |  |  |  |
| Anacardiaceae | *Schmaltzia ovata* (S. Watson) F.A. Barkley |  | 2.7 | 20.3 | 2 | 1 | 7.92 |  |  |  |
| Anacardiaceae | *Toxicodendron vernix* (L.) Shafer |  | -12.3 |  | 2 | 2 | 7.01 |  |  |  |
| Annonaceae | *Annona glabra* L. | **78.890** | 15.8 | 229 | 2 | 1 | 16.15 | 55 | 100–112 | Pirie & Doyle, 2012 |
| Annonaceae | **Asimina triloba* (L.) Dunal |  | -9 | 810 | 2 | 2 | 10.06 |  |  |  |
| Aquifoliaceae | *Ilex ambigua* (Michx.) Torr. | **77.026** | 1.5 |  | 2 | 2 | 6.10 | 56 | 90 (*Ilex*) | Cuénoud *et al*., 2000 |
| Aquifoliaceae | *Ilex amelanchier* M.A. Curtis ex Chapm. |  | 4.7 |  | 2 | 2 | 4.88 |  |  |  |
| Aquifoliaceae | *Ilex cassine* L. |  | 6.8 |  | 2 | 1 | 7.92 |  |  |  |
| Aquifoliaceae | *Ilex coriacea* (Pursh) Chapm. |  | 3.5 |  | 2 | 1 | 6.10 |  |  |  |
| Aquifoliaceae | *Ilex decidua* Walter |  | -4.5 | 6.28 | 2 | 2 | 10.06 |  |  |  |
| Aquifoliaceae | *Ilex krugiana* Loes. Ex Urb. |  | 18.8 |  | 2 | 1 | 10.06 |  |  |  |
| Aquifoliaceae | *Ilex laevigata* (Pursh) A. Gray |  | -9.6 |  | 2 | 2 | 6.10 |  |  |  |
| Aquifoliaceae | *Ilex montana* Torr. & A. Gray |  | -7.6 | 12.73 | 2 | 1 | 11.89 |  |  |  |
| Aquifoliaceae | **Ilex opaca* Aiton |  | -3.1 | 17.2 | 2 | 1 | 14.94 |  |  |  |
| Aquifoliaceae | *Ilex verticillata* (L.) A. Gray |  | -16.6 | 4.97 | 2 | 2 | 7.92 |  |  |  |
| Aquifoliaceae | *Ilex vomitoria* Aiton |  | 3.3 | 10.5 | 2 | 1 | 7.92 |  |  |  |
| Araliaceae | *Aralia spinosa* L. | 69.439 | -6.7 | 3.55 | 2 | 2 | 7.01 |  |  |  |
| Arecaceae | *Acoelorrhaphe wrightii* (Griseb. & H. Wendl.) H. Wendl. ex Becc. | 106.823 | 18 |  | 2 | 1 | 7.92 | **103** | 85–89 | Harly, 2006 |
| Arecaceae | **Coccothrinax argentata* (Jacq.) L.H. Bailey |  | 12.7 |  | 2 | 1 | 8.23 |  |  |  |
| Arecaceae | **Leucothrinax morrisii* (H. Wendl.) C. Lewis & Zona |  | 19.6 |  | 2 | 1 | 12.19 |  |  |  |
| Arecaceae | **Roystonea regia* (Kunth) O.F. Cook |  | 17.8 | 381.1 | 2 | 1 | 40.23 |  |  |  |
| Arecaceae | **Sabal mexicana* Mart. |  | 15.9 |  | 2 | 1 | 15.24 |  |  |  |
| Arecaceae | *Sabal minor* (Jacq.) Pers. |  | 4 |  | 2 | 1 | 3.05 |  |  |  |
| Arecaceae | *Sabal palmetto* (Walter) Lodd. ex Schult. & Schult.f. |  | 7 | 313.95 | 2 | 1 | 24.99 |  |  |  |
| Arecaceae | *Serenoa repens* (W.Bartram) Small |  | 8.2 | 419.6 | 2 | 1 | 7.01 |  |  |  |
| Arecaceae | **Thrinax radiata* Lodd. ex Schult. & Schult.f. |  | 18.9 |  | 2 | 1 | 10.06 |  |  |  |
| Arecaceae | *Washingtonia filifera* (Linden ex André) H. Wendl. Ex de Bary |  | 6.4 |  | 2 | 1 | 15.24 |  |  |  |
| Asparagaceae | *Yucca aloifolia* L. | 43.800 | 7 |  | 2 | 1 | 5.00 |  |  |  |
| Asparagaceae | *Yucca brevifolia* Engelm. |  | -0.3 | 92 | 2 | 1 | 15.24 |  |  |  |
| Asparagaceae | *Yucca carnerosana* (Trel.) McKelvey |  | 5.5 |  |  | 1 | 6.10 |  |  |  |
| Asparagaceae | *Yucca elata* (Engelm.) Engelm. |  | -2.8 | 18.9 | 3 | 1 | 5.00 |  |  |  |
| Asparagaceae | *Yucca faxoniana* Sarg. |  | 4.9 | 121 |  | 1 | 15.24 |  |  |  |
| Asparagaceae | *Yucca gloriosa* L. |  | 6.2 |  | 2 | 1 | 2.99 |  |  |  |
| Asparagaceae | *Yucca mohavensis* Sarg. |  | -0.1 | 145 |  | 1 | 6.10 |  |  |  |
| Asparagaceae | *Yucca rostrata* Engelm. ex Trel. |  | 6.6 |  |  | 1 | 3.96 |  |  |  |
| Asparagaceae | *Yucca schottii* Engelm. |  | 3.1 |  | 2 | 1 | 7.01 |  |  |  |
| Asparagaceae | *Yucca treculeana* Carrière |  | 10.1 |  |  | 1 | 8.02 |  |  |  |
| Asteraceae | *Artemisia tridentata* Nutt. | 42.307 | -12.4 | 0.192 | 1 | 2 | 6.10 |  |  |  |
| Betulaceae | *Alnus incana* (Du Roi) R.T. Clausen | **33.219** | -30.7 | 1.17 | 3 | 2 | 10.06 | 29 | 86–90 | Forest *et al*., 2005 |
| Betulaceae | *Alnus maritima* (Marshall) Muhl. ex Nutt. |  | 0 |  | 3 | 2 | 10.06 |  |  |  |
| Betulaceae | **Alnus oblongifolia* Torr. |  | -1.9 | 2.06 | 3 | 2 | 10.06 |  |  |  |
| Betulaceae | **Alnus rhombifolia* Nutt. |  | -8.4 | 0.548 | 3 | 2 | 20.12 |  |  |  |
| Betulaceae | **Alnus rubra* Bong. |  | -15.7 | 0.7 | 3 | 2 | 40.20 |  |  |  |
| Betulaceae | *Alnus serrulata* (Aiton) Willd. |  | -11.5 |  | 3 | 2 | 3.66 |  |  |  |
| Betulaceae | *Alnus viridis* (Regel) Á.Löve & D. Löve |  | -30.1 |  | 3 | 2 | 10.06 |  |  |  |
| Betulaceae | **Betula alleghaniensis* Britton |  | -18.5 | 0.9 | 3 | 2 | 21.95 |  |  |  |
| Betulaceae | **Betula lenta* L. |  | -15.7 | 0.72 | 3 | 2 | 18.29 |  |  |  |
| Betulaceae | **Betula nigra* L. |  | -11.4 | 1.21 | 3 | 2 | 24.99 |  |  |  |
| Betulaceae | **Betula occidentalis* Hook. |  | -22.9 | 0.18 | 3 | 2 | 12.19 |  |  |  |
| Betulaceae | **Betula papyrifera* Marshall |  | -31.7 | 0.3 | 3 | 2 | 24.99 |  |  |  |
| Betulaceae | *Betula populifolia* Marshall |  | -14.9 | 0.1 | 3 | 2 | 10.06 |  |  |  |
| Betulaceae | **Carpinus caroliniana* Walter |  | -16 | 18.3 | 3 | 2 | 7.92 |  |  |  |
| Betulaceae | *Ostrya knowltonii* Sarg. |  | -5.7 |  | 3 | 2 | 12.19 |  |  |  |
| Betulaceae | **Ostrya virginiana* (Mill) K. Koch |  | -18.5 | 15.41 | 3 | 2 | 12.19 |  |  |  |
| Bignoniaceae | *Amphitecna latifolia* (Mill.) A.H. Gentry | **45.248** | 19.6 |  | (water?) | 1 | 6.10 | 36 | 49–50 | Wehr & Hopkins, 1994 |
| Bignoniaceae | **Catalpa bignonioides* Walter |  | 5.3 | 23.3 | 3 | 2 | 14.94 |  |  |  |
| Bignoniaceae | **Catalpa speciosa* (Warder ex Barney) Warder ex Engelm. |  | -4.1 | 19.57 | 3 | 2 | 29.87 |  |  |  |
| Bignoniaceae | *Chilopsis linearis* (Cav.) Sweet |  | -0.8 | 6.5 | 3 | 2 | 10.06 |  |  |  |
| Boraginaceae | *Bourreria ovata* Miers | 85.473 | 18.9 |  | 2 | 1 | 11.89 | **36** | 33–55 | Miller, 2002 |
| Boraginaceae | *Cordia boissieri* A. DC. |  | 7.9 | 552.76 | 2 | 1 | 11.89 |  |  |  |
| Boraginaceae | **Cordia sebestena* L. |  | 18.9 | 333.33 | 1 | 1 | 9.14 |  |  |  |
| Boraginaceae | **Ehretia anacua* (Terán & Berland.) I.M. Johnst. |  | 7.4 | 42.23 | 2 | 1 | 14.94 |  |  |  |
| Burseraceae | *Bursera fagaroides* (Kunth) Engl. | **42.881** | 5.7 |  | 2 | 2 | 4.88 | 64 | 50–56.5 | Weeks *et al*., 2005 |
| Burseraceae | *Bursera microphylla* A. Gray |  | 10 | 32.46 | 2 | 2 | 5.00 |  |  |  |
| Burseraceae | **Bursera simaruba* (L.) Sarg. |  | 15.5 | 93.2 | 2 | 2 | 19.81 |  |  |  |
| Cactaceae | *Carnegiea gigantea* (Engelm.) Britton & Rose | 28.063 | 5.3 | 1.1 | 2 | 1 | 15.24 |  |  |  |
| Cactaceae | *Cereus thurberi* Engelm. |  | 4.1 |  | 2 | 1 | 6.10 |  |  |  |
| Cactaceae | *Cylindropuntia fulgida* (Engelm.) F.M. Knuth |  | 4.9 | 5.17 | 2 | 1 | 3.05 |  |  |  |
| Canellaceae | *Canella winterana* (L.) Gaertn. | 106.765 | 18.9 |  | 2 | 1 | 10.06 |  |  |  |
| Cannabaceae | *Celtis ehrenbergiana* (Klotzsch) Liebm. | 41.480 | 2.7 | 27.7 | 2 | 2 | 4.88 | **51** | 65-70 | Friis *et al*., 2011 |
| Cannabaceae | **Celtis laevigata* Willd. |  | -5.5 | 83.89 | 2 | 2 | 28.04 |  |  |  |
| Cannabaceae | *Celtis lindheimeri* Engelm. ex K. Koch |  | 10.2 |  | 2 | 2 | 14.02 |  |  |  |
| Cannabaceae | **Celtis occidentalis* L. |  | -18.6 | 108.8 | 2 | 2 | 18.29 |  |  |  |
| Cannabaceae | *Celtis tenuifolia* Nutt. |  | -5.8 |  | 2 | 2 | 10.06 |  |  |  |
| Cannabaceae | *Trema lamarckiana* (Roem. & Schult.) Blume |  | 19.2 |  | 2 | 1 | 6.10 |  |  |  |
| Cannabaceae | *Trema micrantha* (L.) Blume |  | 15.4 | 3.4 | 2 | 1 | 24.99 |  |  |  |
| Capparaceae | *Capparis cynophallophora* L. | 50.503 | 15.4 | 54 | 2 | 1 | 6.10 |  |  |  |
| Capparaceae | *Koeberlinia spinosa* Zucc. |  | 1.9 |  | 2 | 2 | 3.96 |  |  |  |
| Celastraceae | *Canotia holacantha* Torr. | 41.983 | -0.2 | 5.49 | 3 | 2 | 6.10 |  |  |  |
| Celastraceae | *Crossopetalum rhacoma* Crantz |  | 16 | 22.12 | 2 | 1 | 6.10 |  |  |  |
| Celastraceae | *Euonymus atropurpureus* Jacq. |  | -14.1 | 26.43 | 2 | 2 | 8.02 |  |  |  |
| Celastraceae | *Euonymus occidentalis* Nutt. ex. Torr. |  | -0.4 | 18.884 | 2 | 2 | 6.10 |  |  |  |
| Celastraceae | *Maytenus phyllanthoides* Benth. |  | 15 |  | (water) | 1 | 6.10 |  |  |  |
| Celastraceae | *Schaefferia frutescens* Jacq. |  | 19.7 |  | 2 | 1 | 12.19 |  |  |  |
| Chrysobalanaceae | *Chrysobalanus icaco* L. | 63.027 | 17.6 | 2505 | 2 | 1 | 5.18 |  |  |  |
| Clethraceae | *Clethra acuminata* Michx. | 78.134 | -2.2 |  | 3 | 2 | 6.10 |  |  |  |
| Combretaceae | **Conocarpus erectus* L. | 74.034 | 13.7 | 3.3 | 1 | 1 | 20.12 | 83 | 70–90 | Stevens, 2001 |
| Combretaceae | **Laguncularia racemosa* (L.) C.F. Gaertn. |  | 13 | 280 | (water) | 1 | 17.98 |  |  |  |
| Cornaceae | *Cornus alternifolia* L.f. | 96.335 | -19.2 | 43.3 | 2 | 2 | 9.14 | 87 | --- | --- |
| Cornaceae | *Cornus drummondii* C.A. Mey. |  | -9.8 | 25.61 | 2 | 2 | 3.96 |  |  |  |
| Cornaceae | **Cornus florida* L. |  | -8 | 76.9 | 2 | 2 | 14.94 |  |  |  |
| Cornaceae | *Cornus foemina* Mill. |  | -3.9 |  | 2 | 2 | 4.57 |  |  |  |
| Cornaceae | *Cornus glabrata* Benth. |  | -2.8 | 37.726 | 2 | 2 | 6.10 |  |  |  |
| Cornaceae | **Cornus nuttallii* Audubon ex Torr. & A. Gray |  | -9.8 | 85.2 | 2 | 2 | 29.87 |  |  |  |
| Cornaceae | *Cornus sericea* L. |  | -31.6 | 25.8 | 2 | 2 | 4.88 |  |  |  |
| Cornaceae | **Nyssa aquatica* L. |  | -2.5 | 895.59 | (water) | 2 | 35.05 |  |  |  |
| Cornaceae | **Nyssa ogeche* Bartram ex Marshall |  | 8.1 | 337.8 | 2 | 2 | 17.98 |  |  |  |
| Cornaceae | **Nyssa sylvatica* Marshall |  | -10.6 | 125 | 2 | 2 | 39.93 |  |  |  |
| Cyrillaceae | *Cliftonia monophylla* (Lam.) Britton | 76.806 | 8.2 |  | 3 | 1 | 7.92 |  |  |  |
| Cyrillaceae | *Cyrilla racemiflora* L. |  | 2.4 |  | 2 | 1 | 7.92 |  |  |  |
| Ebenaceae | **Diospyros texana* Scheele | 88.362 | 6.4 | 147.8 | 2 | 1 | 16.15 | 77 | 65–99 | Basinger & Christophel, 1985 |
| Ebenaceae | **Diospyros virginiana* L. |  | -5.4 | 349.9 | 2 | 1 | 20.12 |  |  |  |
| Elaeagnaceae | *Lepargyrea argentea* (Pursh) Greene | 64.039 | -18.7 | 6.63 | 2 | 2 | 4.88 |  |  |  |
| Ericaceae | **Arbutus arizonica* (A. Gray) Sarg. | **76.806** | 4.7 |  | 2 | 1 | 15.24 | 44 | 65–69 | Muller, 1981; Jordan *et al*., 2010 |
| Ericaceae | **Arbutus menziesii* Pursh |  | -3.4 | 3.3 | 2 | 1 | 12.19 |  |  |  |
| Ericaceae | **Arbutus texana* Buckley |  | 3.8 |  | 2 | 1 | 7.92 |  |  |  |
| Ericaceae | *Batodendron arboreum* Nutt. |  | -3 | 1.2 | 2 | 2 | 8.53 |  |  |  |
| Ericaceae | *Chamaedaphne latifolia* (L.) Kuntze |  | -8.2 | 0.02 | 3 | 1 | 9.14 |  |  |  |
| Ericaceae | *Elliottia racemosa* Muhl. ex Elliott |  | 6.1 |  |  | 2 | 10.06 |  |  |  |
| Ericaceae | *Hymenanthes catawbiensis* (Michx.) H.F. Copel. |  | -2.8 | 0.07 | 3 | 1 | 6.10 |  |  |  |
| Ericaceae | *Hymenanthes macrophylla* (D. Don ex G. Don) H.F. Copel. |  | -8.2 | 0.27 | 3 | 1 | 7.62 |  |  |  |
| Ericaceae | *Hymenanthes maxima* (L.) H.F. Copel. |  | -11.1 | 0.09 | 1 | 1 | 12.19 |  |  |  |
| Ericaceae | **Oxydendrum arboreum* (L.) DC. |  | -5 | 0.18 | 3 | 2 | 18.29 |  |  |  |
| Ericaceae | *Xolisma ferruginea* (Walter) A. Heller |  | 8.1 |  | 3 | 1 | 9.14 |  |  |  |
| Euphorbiaceae | *Gymnanthes lucida* Sw. | **57.380** | -11.8 |  | 2 | 1 | 10.06 | 85 | 55–65 | Muller, 1981 |
| Euphorbiaceae | **Hippomane mancinella* L. |  | 18.9 |  | 2 | 1 | 11.89 |  |  |  |
| Euphorbiaceae | *Sebastiania bilocularis* S. Watson |  | 10.1 |  | 1 | 2 | 6.10 |  |  |  |
| Fabaceae | **Acacia farnesiana* (L.) Willd. | **63.803** | 7.6 | 72.5 | 2 | 2 | 10.06 | 73 | 56 | Herendeen *et al*., 1992 |
| Fabaceae | *Acacia greggii* A. Gray |  | 0.1 | 120.3 | 2 | 2 | 7.01 |  |  |  |
| Fabaceae | *Acacia macracantha* Willd. |  | 20.3 | 35.52 | 2 | 2 | 7.01 |  |  |  |
| Fabaceae | *Acacia tortuosa* (L.) Willd. |  | 8.6 | 59.52 | 2 | 2 | 6.10 |  |  |  |
| Fabaceae | *Acacia wrightii* A. Gray |  | 6.4 | 63.18 | 2 | 2 | 3.05 |  |  |  |
| Fabaceae | *Caesalpinia mexicana* A. Gray |  | 13.2 | 46.83 | 2 | 1 | 10.06 |  |  |  |
| Fabaceae | *Cercidium floridum* A. Gray |  | -2 | 229 | 1 | 2 | 10.06 |  |  |  |
| Fabaceae | *Cercidium macrum* I.M. Johnst. |  | -2.2 | 82.383 | 1 | 2 | 7.92 |  |  |  |
| Fabaceae | *Cercidium microphyllum* (Torr.) Rose & I.M. Johnst. |  | -8 | 139.87 | 1 | 2 | 6.10 |  |  |  |
| Fabaceae | **Cercis canadensis* L. |  | 5.5 | 23.9 | 3 | 2 | 7.92 |  |  |  |
| Fabaceae | *Cercis occidentalis* A. Gray |  | -11.8 | 37 | 3 | 2 | 7.01 |  |  |  |
| Fabaceae | **Cladrastis kentukea* (Dum. Cours.) Rudd |  | -1.8 |  | 3 | 2 | 18.29 |  |  |  |
| Fabaceae | **Ebenopsis ebano* (Berland.) Barney & J.W. Grimes |  | 13 | 566.99 | 1 | 1 | 9.14 |  |  |  |
| Fabaceae | *Erythrina flabelliformis* Kearney |  | 3.2 | 861 | 2 | 2 | 4.57 |  |  |  |
| Fabaceae | *Erythrina herbacea* L. |  | 5.8 | 283.5 | 2 | 2 | 4.88 |  |  |  |
| Fabaceae | *Eysenhardtia polystachya* (Ortega) Sarg. |  | 5.7 | 8.73 | 3 | 1 | 7.01 |  |  |  |
| Fabaceae | **Gleditsia aquatica* Marshall |  | -2.4 |  | (water) | 2 | 15.24 |  |  |  |
| Fabaceae | **Gleditsia triacanthos* L. |  | -10.7 | 181.3 | 2 | 2 | 30.48 |  |  |  |
| Fabaceae | **Gymnocladus dioica* (L.) K. Koch |  | 18.4 | 1843 | 1 | 2 | 30.48 |  |  |  |
| Fabaceae | *Havardia pallens* (Benth.) Britton & Rose |  | 10.2 | 41.4 | 2 | 1 | 10.06 |  |  |  |
| Fabaceae | *Leucaena leucocephala* (Lam.) de Wit |  | 5.6 | 48 | 1 | 1 | 10.06 |  |  |  |
| Fabaceae | *Leucaena pulverulenta* (Schltdl.) Benth. |  | 16.7 |  | 1 | 1 | 15.24 |  |  |  |
| Fabaceae | *Leucaena retusa* Benth. |  | 8 | 55 | 1 | 1 | 7.92 |  |  |  |
| Fabaceae | *Lysiloma latisiliquum* (L.) Benth. |  | 17.8 | 21.65 | 3 | 2 | 20.12 |  |  |  |
| Fabaceae | **Olneya tesota* A. Gray |  | 6.4 | 204.05 | 2 | 1 | 10.06 |  |  |  |
| Fabaceae | *Parkinsonia aculeata* L. |  | 6.1 | 89.7 | 1 | 1 | 12.19 |  |  |  |
| Fabaceae | **Piscidia piscipula* (L.) Sarg. |  | 17.4 |  | 2 | 2 | 15.24 |  |  |  |
| Fabaceae | *Pithecellobium keyense* Coker |  | 17.6 |  | 2 | 1 | 6.10 |  |  |  |
| Fabaceae | *Pithecellobium unguis-cati* (L.) Benth. |  | 17.1 | 88.77 | 2 | 1 | 7.32 |  |  |  |
| Fabaceae | **Prosopis juliflora* (Sw) DC. |  | 1.8 | 56 | 2 | 1 | 10.06 |  |  |  |
| Fabaceae | **Prosopis pubescens* Benth. |  | -1.7 | 9.24 | 2 | 2 | 10.06 |  |  |  |
| Fabaceae | *Psorothamnus spinosus* (A. Gray) Barneby |  | 6.2 | 14.12 | 1 | 2 | 7.92 |  |  |  |
| Fabaceae | *Robinia hispida* L. |  | -0.8 | 16.54 | 1 | 2 | 4.57 |  |  |  |
| Fabaceae | **Robinia neomexicana* A. Gray |  | -6 | 20.5 | 1 | 2 | 7.92 |  |  |  |
| Fabaceae | **Robinia pseudoacacia* L. |  | -5.1 | 20.4 | 1 | 2 | 18.29 |  |  |  |
| Fabaceae | *Robinia viscosa* Vent. |  | -0.4 | 25.45 | 1 | 2 | 10.06 |  |  |  |
| Fabaceae | **Sophora affinis* Torr. & A. Gray |  | 3.1 | 73.05 | 1 | 1 | 10.67 |  |  |  |
| Fabaceae | *Sophora secundiflora* (Ortega) DC. |  | 5.7 | 672.5 | 1 | 1 | 10.97 |  |  |  |
| Fagaceae | **Castanea dentata* (Marshall) Borkh. | **63.810** | -3.9 | 3467.3 | 2 | 2 | 35.05 | 55 | 83–86 | Taylor *et al*., 1993 |
| Fagaceae | **Castanea ozarkensis* Ashe |  | -9.5 |  | 2 | 2 | 19.99 |  |  |  |
| Fagaceae | **Castanea pumila* (L.) Mill. |  | -0.4 |  | 2 | 2 | 7.92 |  |  |  |
| Fagaceae | **Chrysolepis chrysophylla* (Douglas ex Hook.) Hjelmq. |  | -3.6 | 470.04 | 2 | 1 | 40.23 |  |  |  |
| Fagaceae | **Fagus grandifolia* Ehrh. |  | -14.5 | 266 | 2 | 2 | 30.48 |  |  |  |
| Fagaceae | **Lithocarpus densiflorus* (Hook. & Arn.) Rehder |  | 1.2 | 5929.5 | 2 | 1 | 30.48 |  |  |  |
| Fagaceae | **Quercus agrifolia* Née |  | 5.6 | 2270.48 | 2 | 1 | 24.99 |  |  |  |
| Fagaceae | *Quercus ajoensis* C.H. Mull. |  | 10.9 |  | 2 | 1 | 10.06 |  |  |  |
| Fagaceae | **Quercus alba* L. |  | -14.1 | 2997 | 2 | 2 | 35.05 |  |  |  |
| Fagaceae | **Quercus arizonica* Sarg. |  | -1.1 |  | 2 | 1 | 20.12 |  |  |  |
| Fagaceae | *Quercus arkansana* Sarg. |  | 3.9 |  | 2 | 2 | 24.99 |  |  |  |
| Fagaceae | *Quercus austrina* Small |  | 5.5 |  | 2 | 2 | 35.05 |  |  |  |
| Fagaceae | **Quercus bicolor* Willd. |  | -14.3 | 3459 | 2 | 2 | 35.05 |  |  |  |
| Fagaceae | **Quercus canbyi* Trel. |  | 8.4 |  | 2 | 1 | 7.99 |  |  |  |
| Fagaceae | *Quercus chapmanii* Sarg. |  | 9.3 |  | 2 | 1 | 7.92 |  |  |  |
| Fagaceae | **Quercus chrysolepis* Liebm. |  | -2.7 | 3220.26 | 2 | 1 | 30.48 |  |  |  |
| Fagaceae | **Quercus coccinea* Münchh. |  | -8 | 1931 | 2 | 2 | 30.48 |  |  |  |
| Fagaceae | **Quercus douglasii* Hook. & Arn. |  | 0.1 | 4540.46 | 2 | 2 | 20.12 |  |  |  |
| Fagaceae | *Quercus dumosa* Nutt. |  | 1.3 | 3170 | 2 | 1 | 3.05 |  |  |  |
| Fagaceae | **Quercus ellipsoidalis* E.J.Hill |  | -17.1 | 1557.93 | 2 | 2 | 20.12 |  |  |  |
| Fagaceae | **Quercus emoryi* Torr. |  | 1.5 | 191 | 2 | 1 | 20.12 |  |  |  |
| Fagaceae | **Quercus engelmanni*i Greene |  | 5.1 |  | 2 | 1 | 18.29 |  |  |  |
| Fagaceae | **Quercus falcata* Michx. |  | -1.8 | 840.99 | 2 | 2 | 30.48 |  |  |  |
| Fagaceae | **Quercus gambelii* Nutt. |  | -12.3 | 761 | 2 | 2 | 23.01 |  |  |  |
| Fagaceae | **Quercus garryana* Douglas ex Hook. |  | -5.5 | 4369.36 | 2 | 2 | 20.12 |  |  |  |
| Fagaceae | *Quercus georgiana* M.A. Curtis |  | 5 |  | 2 | 2 | 7.92 |  |  |  |
| Fagaceae | *Quercus glaucoides* M. Martens & Galeotti |  | 7.3 |  | 2 | 2 | 15.24 |  |  |  |
| Fagaceae | **Quercus gravesii* Sudw. |  | 5.6 |  | 2 | 2 | 12.98 |  |  |  |
| Fagaceae | **Quercus grisea* Liebm. |  | -3.5 |  | 2 | 1 | 19.81 |  |  |  |
| Fagaceae | *Quercus havardii* Rydb. |  | 0.4 |  | 2 | 2 | 2.13 |  |  |  |
| Fagaceae | **Quercus hypoleucoides* A. Camus |  | -1.5 |  | 2 | 1 | 12.19 |  |  |  |
| Fagaceae | **Quercus ilicifolia* Wangenh. |  | -11.3 | 648.49 | 2 | 2 | 7.92 |  |  |  |
| Fagaceae | **Quercus imbricaria* Michx. |  | -7.5 | 1093.99 | 2 | 2 | 24.99 |  |  |  |
| Fagaceae | **Quercus incana* Bartram |  | 3.8 | 1000 | 2 | 2 | 12.19 |  |  |  |
| Fagaceae | **Quercus kelloggii* Newb. |  | -2.7 | 4779.8 | 2 | 2 | 28.04 |  |  |  |
| Fagaceae | **Quercus laevis* Walter |  | 3.4 | 1149.67 | 2 | 2 | 15.24 |  |  |  |
| Fagaceae | **Quercus laurifolia* Michx. |  | 2.2 | 810.99 | 2 | 1 | 30.48 |  |  |  |
| Fagaceae | **Quercus lobata* Neé |  | 3.2 | 4551 | 2 | 2 | 30.48 |  |  |  |
| Fagaceae | **Quercus lyrata* Walter |  | -7.5 | 3243.47 | 2 | 2 | 30.48 |  |  |  |
| Fagaceae | **Quercus macrocarpa* Michx. |  | -21.5 | 6145 | 2 | 2 | 40.23 |  |  |  |
| Fagaceae | **Quercus marilandica* (L.) Münchh. |  | -6.6 |  | 2 | 2 | 12.19 |  |  |  |
| Fagaceae | **Quercus michauxii* Nutt. |  | -3.2 | 5340 | 2 | 2 | 39.62 |  |  |  |
| Fagaceae | *Quercus mohriana* Buckley ex Rydb. |  | 0.3 |  | 2 | 1 | 20.12 |  |  |  |
| Fagaceae | **Quercus muehlenbergii* Engelm. |  | -9.7 | 1192.17 | 2 | 2 | 30.48 |  |  |  |
| Fagaceae | *Quercus myrtifolia* Willd. |  | 8.8 |  | 2 | 1 | 12.19 |  |  |  |
| Fagaceae | **Quercus nigra* L. |  | 0.5 | 1139 | 2 | 2 | 24.99 |  |  |  |
| Fagaceae | **Quercus oblongifolia* Torr. |  | 3.1 |  | 2 | 2 | 7.92 |  |  |  |
| Fagaceae | **Quercus oglethorpensis* W.H. Duncan |  | 5.4 |  | 2 | 2 | 19.81 |  |  |  |
| Fagaceae | **Quercus pagoda* Raf. |  | -1 | 783.03 | 2 | 2 | 40.23 |  |  |  |
| Fagaceae | *Quercus palmeri* Engelm. |  | -0.5 |  | 2 | 1 | 3.05 |  |  |  |
| Fagaceae | **Quercus palustris* Münchh. |  | -8.4 | 1087 | 2 | 2 | 24.99 |  |  |  |
| Fagaceae | **Quercus phellos* L. |  | -1.8 | 893.36 | 2 | 2 | 27.43 |  |  |  |
| Fagaceae | **Quercus prinoides* Willd. |  | -9.3 |  | 2 | 2 | 5.18 |  |  |  |
| Fagaceae | *Quercus pungens* Liebm. |  | 1 |  | 2 | 1 | 7.92 |  |  |  |
| Fagaceae | **Quercus rubra* L. |  | -17.6 | 3143 | 2 | 2 | 24.99 |  |  |  |
| Fagaceae | **Quercus rugosa* Neé |  | -1.5 |  | 2 | 1 | 20.12 |  |  |  |
| Fagaceae | **Quercus shumardii* Buckley |  | -5.5 | 4540.46 | 2 | 2 | 30.48 |  |  |  |
| Fagaceae | **Quercus sinuata* Walter |  | 3.6 | 1565.6 | 2 | 2 | 30.48 |  |  |  |
| Fagaceae | **Quercus stellata* Wangenh. |  | -5.7 | 1194.83 | 2 | 2 | 20.12 |  |  |  |
| Fagaceae | *Quercus toumeyi* Sarg. |  | 1.3 |  | 2 | 1 | 2.01 |  |  |  |
| Fagaceae | **Quercus velutina* Lam. |  | -10.7 | 1852 | 2 | 2 | 24.99 |  |  |  |
| Fagaceae | **Quercus virginiana* Mill. |  | 2.6 | 1239 | 2 | 1 | 20.12 |  |  |  |
| Fagaceae | **Quercus wislizeni* A. DC. |  | -2.8 | 3632.37 | 2 | 1 | 9.14 |  |  |  |
| Garryaceae | *Garrya elliptica* Douglas ex Lindl. | 89.592 | 2.7 | 23.06 | 2 | 1 | 6.10 |  |  |  |
| Hammamelidaceae | *Hamamelis virginiana* L. | 101.626 | -13.1 | 46.7 | 2 | 2 | 7.92 |  |  |  |
| Juglandaceae | **Carya alba* (L.) Nutt. ex Elliot | **51.010** | -8.6 | 5148 | 2 | 2 | 35.05 | 32 | >60 | Manos *et al*., 2007 |
| Juglandaceae | **Carya aquatica* (F. Michx.) Nutt. ex Elliott |  | -1.2 | 2771.8 | 2 | 2 | 35.05 |  |  |  |
| Juglandaceae | **Carya cordiformis* (Wangenh.) K. Koch |  | -15.7 | 2797.6 | 2 | 2 | 35.05 |  |  |  |
| Juglandaceae | *Carya floridana* Sarg. |  | 13.6 |  | 2 | 2 | 24.99 |  |  |  |
| Juglandaceae | **Carya glabra* (Mill.) Sweet |  | -9.4 | 3502 | 2 | 2 | 40.23 |  |  |  |
| Juglandaceae | **Carya illinoensis* (Wangenh.) K. Koch |  | -8 | 4142 | 2 | 2 | 60.96 |  |  |  |
| Juglandaceae | **Carya laciniosa* (F. Michx.) G. Don |  | -7.8 | 15141 | 2 | 2 | 40.23 |  |  |  |
| Juglandaceae | **Carya myristiciformis* (F. Michx.) Nutt. ex Elliot |  | 4.1 | 3681 | 2 | 2 | 35.05 |  |  |  |
| Juglandaceae | **Carya ovata* (Mill.) K. Koch |  | -12.2 | 4401 | 2 | 2 | 45.72 |  |  |  |
| Juglandaceae | **Carya pallida* (Ashe) Engelm. & Graebn. |  | -2.2 |  | 2 | 2 | 15.24 |  |  |  |
| Juglandaceae | **Carya texana* Buckley |  | -4.9 |  | 2 | 2 | 28.04 |  |  |  |
| Juglandaceae | **Juglans californica* S. Watson |  | 5.6 | 9081.42 | 2 | 2 | 9.75 |  |  |  |
| Juglandaceae | **Juglans cinerea* L. |  | -14.8 | 14026 | 2 | 2 | 30.48 |  |  |  |
| Juglandaceae | **Juglans hindsii* Jeps. ex R.E. Sm. |  | 6.5 | 9068.04 | 2 | 2 | 19.81 |  |  |  |
| Juglandaceae | **Juglans major* (Torr.) A. Heller |  | -1 | 5019.96 | 2 | 2 | 18.29 |  |  |  |
| Juglandaceae | **Juglans microcarpa* Berland. |  | -1.1 | 4965.18 | 2 | 2 | 15.24 |  |  |  |
| Juglandaceae | **Juglans nigra* L. |  | -12.3 | 11074.8 | 2 | 2 | 40.23 |  |  |  |
| Lauraceae | *Licaria triandra* (Sw.) Koesterm. | **99.807** | 19.4 |  | 2 | 1 | 12.01 | 52 | 93–100 | Drinnan *et al*., 1990 |
| Lauraceae | *Nectandra coriacea* (Sw.) Griseb. |  | 14.7 |  | 2 | 1 | 14.02 |  |  |  |
| Lauraceae | **Persea borbonia* (L.) Spreng. |  | 1.7 |  | 2 | 1 | 20.12 |  |  |  |
| Lauraceae | *Persea palustris* (Raf.) Sarg. |  | 3.1 |  | 2 | 1 | 12.19 |  |  |  |
| Lauraceae | **Sassafras albidum* (Nutt.) Nees |  | -8.9 | 79 | 2 | 1 | 15.24 |  |  |  |
| Lauraceae | **Umbellularia californica* (Hook. & Arn.) Nutt. |  | -0.1 | 1801 | 2 | 1 | 24.99 |  |  |  |
| Magnoliaceae | **Liriodendron tulipifera* L. | **94.250** | -8.4 | 25.8 | 3 | 2 | 50.29 | 56 | 100–112 | Pirie & Doyle, 2012 |
| Magnoliaceae | **Magnolia acuminata* (L.) L. |  | -7 | 88.51 | 2 | 2 | 30.48 |  |  |  |
| Magnoliaceae | **Magnolia fraseri* Walter |  | -4.1 | 99.85 | 2 | 2 | 15.24 |  |  |  |
| Magnoliaceae | **Magnolia grandiflora* (L.) |  | 5.4 | 87 | 2 | 1 | 30.48 |  |  |  |
| Magnoliaceae | **Magnolia macrophylla* Michx. |  | -1.6 | 167.8 | 2 | 2 | 15.24 |  |  |  |
| Magnoliaceae | **Magnolia tripetala* (L.) L. |  | -3.6 | 263.16 | 2 | 2 | 15.24 |  |  |  |
| Magnoliaceae | **Magnolia virginiana* L. |  | -2.9 | 58.48 | 2 | 1 | 24.99 |  |  |  |
| Malpighiaceae | *Byrsonima lucida* (Mill.) DC. | 73.493 | 19 |  | 2 | 1 | 6.10 |  |  |  |
| Malvaceae | *Fremontodendron californicum* (Torr.) Coult. | 65.834 | 3.2 | 28.4 | 3 | 1 | 9.14 | 67 | >73 | Hensley, 2009 |
| Malvaceae | *Fremontodendron mexicanum* Davidson |  | 10.4 | 20 | 3 | 1 | 6.10 |  |  |  |
| Malvaceae | **Tilia americana* L. |  | -18.4 | 95.5 | 2 | 2 | 24.99 |  |  |  |
| Malvaceae | **Tilia caroliniana* (Small) A.E. Murray |  | -0.6 |  | 2 | 2 | 20.12 |  |  |  |
| Melastomataceae | *Tetrazygia bicolor* (Mill.) Cogn. | 66.476 | 18.8 |  | 2 | 1 | 9.14 |  |  |  |
| Meliaceae | **Swietenia mahagoni* (L.) Jacq. | 49.350 | 18.7 | 110 | 3 | 1 | 14.94 | 50 | 59–70 | Muellner *et al*., 2006 |
| Moraceae | **Ficus aurea* Nutt. | 25.230 | 15.4 |  | 2 | 1 | 20.12 | **51** | >65 | Zerega *et al*., 2005 |
| Moraceae | *Ficus citrifolia* Mill. |  | 18.2 | 0.9 | 2 | 1 | 15.24 |  |  |  |
| Moraceae | **Maclura pomifera* (Raf.) C.K. Schneid. |  | 3.2 | 33 | 2 | 2 | 20.12 |  |  |  |
| Moraceae | **Morus celtidifolia* Kunth |  | -2.9 |  | 2 | 2 | 7.92 |  |  |  |
| Moraceae | **Morus rubra* L. |  | -11.9 | 1.5 | 2 | 2 | 20.12 |  |  |  |
| Myricaceae | *Myrica californica* Cham. & Schltdl. | 51.010 | 1.9 | 21.34 | 2 | 1 | 7.92 |  |  |  |
| Myricaceae | *Myrica cerifera* L. |  | 0.8 | 6.2 | 2 | 1 | 12.19 |  |  |  |
| Myricaceae | *Myrica heterophylla* Raf. |  | -0.9 | 27.5 | 2 | 1 | 2.99 |  |  |  |
| Myricaceae | *Myrica inodora* W. Bartram |  | 8.9 |  | 2 | 1 | 7.01 |  |  |  |
| Myricaceae | *Myrica pensylvanica* Mirb. |  | -11.9 | 8.247 | 2 | 2 | 3.05 |  |  |  |
| Myrtaceae | *Calyptranthes pallens* Griseb. | **80.914** | 17.3 |  | 2 | 1 | 7.92 | 51 | 85–87 | Biffen *et al*., 2010 |
| Myrtaceae | *Calyptranthes zuzygium* (L.) Sw. |  | 18.7 |  | 2 | 1 | 12.19 |  |  |  |
| Myrtaceae | *Eugenia axillaris* (Sw.) Willd. |  | 15.8 | 180.7 | 2 | 1 | 7.92 |  |  |  |
| Myrtaceae | *Eugenia confusa* DC. |  | 18.7 |  | 2 | 1 | 17.98 |  |  |  |
| Myrtaceae | **Eugenia foetida* Pers. |  | 15.8 |  | 2 | 1 | 6.10 |  |  |  |
| Myrtaceae | *Mosiera longipes* (O.Berg) Small |  | 18.8 |  | 2 | 1 | 2.74 |  |  |  |
| Myrtaceae | *Myrcianthes fragrans* (Sw.) McVaugh |  | 14.5 |  | 2 | 1 | 7.92 |  |  |  |
| Nyctaginaceae | **Guapira discolor* (Spreng.) Little | 30.642 | 15.5 |  | 2 | 1 | 15.85 | **38** | >55 | Muller, 1981 |
| Olacaceae | *Schoepfia schreberi*J.F. Gmel. | 114.501 | 14.7 |  |  | 1 | 10.06 |  |  |  |
| Olacaceae | *Ximenia americana* L. |  | 12.3 | 732 | 2 | 1 | 9.14 |  |  |  |
| Oleaceae | *Chionanthus virginicus* L. | 58.888 | -3.4 | 462.03 | 2 | 2 | 10.06 | 74 | --- | --- |
| Oleaceae | *Forestiera acuminata* (Michx.) Poir. |  | -4.6 |  | 2 | 1 | 10.06 |  |  |  |
| Oleaceae | *Forestiera angustifolia* Torr. |  | 6.3 |  | 2 | 1 | 7.01 |  |  |  |
| Oleaceae | *Forestiera phillyreoides* (Benth.) Torr. |  | 5.7 |  | 2 | 1 | 7.92 |  |  |  |
| Oleaceae | *Forestiera segregata* (Jacq.) Krug & Urb. |  | 9.3 |  | 2 | 1 | 6.10 |  |  |  |
| Oleaceae | **Fraxinus americana* L. |  | -15.8 | 38.1 | 3 | 2 | 24.99 |  |  |  |
| Oleaceae | *Fraxinus anomala* Torr. ex S. Watson |  | 0.6 | 19.27 | 3 | 2 | 7.92 |  |  |  |
| Oleaceae | *Fraxinus berlandieriana* A. DC. |  | 6.9 |  | 3 | 2 | 9.14 |  |  |  |
| Oleaceae | **Fraxinus caroliniana* Mill. |  | 0.6 | 77.93 | 3 | 2 | 11.89 |  |  |  |
| Oleaceae | *Fraxinus cuspidata* Torr. |  | -4.3 |  | 3 | 2 | 7.92 |  |  |  |
| Oleaceae | *Fraxinus dipetala* Hook. & Arn. |  | -4.4 | 36 | 3 | 2 | 7.01 |  |  |  |
| Oleaceae | *Fraxinus gooddingii* Little |  | 6.1 | 9.66 | 3 | 1 | 7.62 |  |  |  |
| Oleaceae | *Fraxinus greggii* A. Gray |  | 8.4 |  | 3 | 1 | 7.92 |  |  |  |
| Oleaceae | **Fraxinus latifolia* Benth. |  | -1.3 | 46.3 | 3 | 2 | 24.99 |  |  |  |
| Oleaceae | **Fraxinus nigra* Marshall |  | -20.8 | 44.4 | 3 | 2 | 20.12 |  |  |  |
| Oleaceae | *Fraxinus papillosa* Lingelsh. |  | 3.1 |  | 3 | 1 | 6.10 |  |  |  |
| Oleaceae | **Fraxinus pennsylvanica* Marshall |  | -23 | 32.3 | 3 | 2 | 20.12 |  |  |  |
| Oleaceae | **Fraxinus profunda* (Bush) Bush |  | -5 | 142.32 | 3 | 2 | 29.87 |  |  |  |
| Oleaceae | **Fraxinus quadrangulata* Michx. |  | -8.1 | 71.4 | 3 | 2 | 20.12 |  |  |  |
| Oleaceae | **Fraxinus texensis* (A. Gray) Sarg. |  | 3.9 |  | 3 | 2 | 16.15 |  |  |  |
| Oleaceae | **Fraxinus velutina* Torr. |  | -3 | 19.63 | 3 | 2 | 11.89 |  |  |  |
| Oleaceae | *Osmanthus americanus* (L.) A. Gray |  | 4.6 |  | 2 | 1 | 14.94 |  |  |  |
| Picramniaceae | *Alvaradoa amorphoides* Liebm. | 103.862 | 18.8 | 7.9 | 3 | 1 | 14.94 |  |  |  |
| Platanaceae | **Platanus occidentalis* L. | 126.136 | -9.9 | 2.83 | 3 | 2 | 35.05 | 99 | 105-113 | Hickey & Doyle, 1977; Friis *et al*., 2011 |
| Platanaceae | **Platanus racemosa* Nutt. |  | 3.2 | 3.42 | 3 | 2 | 30.48 |  |  |  |
| Polygonaceae | **Coccoloba diversifolia* Jacq. | 59.247 | 18.7 |  | 2 | 1 | 20.12 | 60 | 55–65 | Muller, 1981 |
| Polygonaceae | *Coccoloba uvifera* (L.) L. |  | 15.9 |  | 2 | 1 | 15.24 |  |  |  |
| Primulaceae | *Ardisia escallonioides* Schltdl. & Cham. | 36.689 | 14.7 |  | 2 | 1 | 7.50 |  |  |  |
| Primulaceae | *Jacquinia keyensis* Mez |  | 17.1 |  |  | 1 | 6.10 |  |  |  |
| Primulaceae | *Myrsine cubana* A. DC. |  | 13 |  | 2 | 1 | 6.10 |  |  |  |
| Putranjivaceae | *Drypetes lateriflora* (Sw.) Krug & Urb. | 73.589 | 16 |  | 2 | 1 | 10.06 |  |  |  |
| Rhamnaceae | *Ceanothus spinosus* Nutt | 64.039 | 5.4 | 9.2 | 3 | 1 | 6.10 | **71** | 72-75 | Calvillo-Candell & Cevallos-Ferriz, 2007 |
| Rhamnaceae | *Ceanothus thyrsiflorus* Eschw. |  | 3.7 | 2.51 | 3 | 1 | 6.10 |  |  |  |
| Rhamnaceae | *Colubrina arborescens* (Mill.) Sarg. |  | 18.8 | 13.76 | 1 | 1 | 7.92 |  |  |  |
| Rhamnaceae | **Colubrina cubensis* (Jacq.) Brongn. |  | 18.8 |  | 1 | 1 | 9.14 |  |  |  |
| Rhamnaceae | *Condalia globosa* I.M. Johnst. |  | 7.7 |  | 2 | 1 | 6.10 |  |  |  |
| Rhamnaceae | **Condalia hookeri* M.C. Johnst. |  | 7.7 | 17.115 | 2 | 1 | 9.14 |  |  |  |
| Rhamnaceae | *Frangula betulifolia* (Greene) Grubov |  | -2.7 | 48.62 | 2 | 2 | 6.10 |  |  |  |
| Rhamnaceae | *Frangula californica* (Eschsch.) A. Gray |  | -2.8 | 103.2 | 2 | 1 | 6.10 |  |  |  |
| Rhamnaceae | *Frangula caroliniana* (Walter) A. Gray |  | -2.5 |  | 2 | 2 | 11.89 |  |  |  |
| Rhamnaceae | *Frangula purshiana* Cooper |  | -9.5 | 33.6 | 2 | 2 | 11.89 |  |  |  |
| Rhamnaceae | *Krugiodendron ferreum* (Vahl) Urb. |  | 17.5 |  | 2 | 1 | 9.14 |  |  |  |
| Rhamnaceae | *Rhamnus crocea* Nutt. |  | -2.3 | 7.2 | 2 | 1 | 7.92 |  |  |  |
| Rhizophoraceae | **Rhizophora mangle* L. | 76.371 | 15.8 | 10100 | (water) | 1 | 24.99 | **63** | 45–60 | Graham, 2006 |
| Rosaceae | **Amelanchier arborea* (F. Michx) Fernald | **72.997** | -18.3 | 5.67 | 2 | 2 | 12.19 | 85 | 45–55 | DeVore & Pigg, 2007 |
| Rosaceae | *Amelanchier interior* E.L. Nielsen |  | -16.1 |  | 2 | 2 | 7.92 |  |  |  |
| Rosaceae | **Amelanchier sanguinea* (Pursh) DC. |  | -17.8 | 5.4 | 2 | 2 | 6.10 |  |  |  |
| Rosaceae | **Armeniaca dasycarpa* (Ehrh.) Borkh. |  | -19.4 |  | 2 | 2 | 10.06 |  |  |  |
| Rosaceae | **Aucuparia americana* (Marshall) Nieuwl. |  | -22.4 | 3.17 | 2 | 2 | 9.14 |  |  |  |
| Rosaceae | *Cercocarpus betuloides* Nutt. |  | 11.3 | 16.6 | 3 | 1 | 7.92 |  |  |  |
| Rosaceae | *Cercocarpus breviflorus* A. Gray |  | 2.6 | 7.77 | 3 | 1 | 7.01 |  |  |  |
| Rosaceae | **Cercocarpus ledifolius* Nutt. |  | -2 | 10.55 | 3 | 1 | 12.19 |  |  |  |
| Rosaceae | *Cowania mexicana* D. Don |  | -7.4 | 7.09 | 3 | 1 | 7.92 |  |  |  |
| Rosaceae | *Crataegus aestivalis* (Walter) Torr. & A.Gray |  | 5.5 |  | 2 | 2 | 8.84 |  |  |  |
| Rosaceae | *Crataegus coccinoides* Ashe |  | -1.4 |  | 2 | 2 | 4.57 |  |  |  |
| Rosaceae | *Crataegus columbiana* Howell |  | -17.3 | 42.55 | 2 | 2 | 12.19 |  |  |  |
| Rosaceae | **Crataegus crus-galli* L. |  | -12.5 | 90.4 | 2 | 2 | 9.14 |  |  |  |
| Rosaceae | *Crataegus douglasii* Lindl. |  | -17.8 | 23.13 | 2 | 2 | 12.19 |  |  |  |
| Rosaceae | *Crataegus erythropoda* Ashe |  | -12 |  | 2 | 2 | 4.88 |  |  |  |
| Rosaceae | *Crataegus flabellata* (Bosc ex Spach) Rydb. |  | -13.8 |  | 2 | 2 | 6.00 |  |  |  |
| Rosaceae | *Crataegus intricata* Lange |  | -10.3 |  | 2 | 2 | 7.62 |  |  |  |
| Rosaceae | *Crataegus phaenopyrum* (L.f.) Medik. |  | -1.4 | 53.47 | 2 | 2 | 12.19 |  |  |  |
| Rosaceae | *Crataegus pruinosa* (H.L. Wendl.) K. Koch |  | -14.3 | 50.433 | 2 | 2 | 6.10 |  |  |  |
| Rosaceae | *Crataegus saligna* Greene |  | -12.3 |  | 2 | 2 | 6.10 |  |  |  |
| Rosaceae | *Crataegus tracyi* Ashe ex Eggl. |  | 5.6 |  | 2 | 2 | 6.10 |  |  |  |
| Rosaceae | *Crataegus uniflora* Münchh. |  | -4.8 | 39.1 | 2 | 2 | 4.88 |  |  |  |
| Rosaceae | *Crataegus viridis* L. |  | -1.2 |  | 2 | 2 | 9.14 |  |  |  |
| Rosaceae | *Emplectocladus fremontii* (S. Watson) Dayton |  | 5.7 | 134.3 | 2 | 2 | 5.18 |  |  |  |
| Rosaceae | *Heteromeles salicifolia* (C. Presl) Abrams |  | 1 | 11.4 | 2 | 1 | 6.10 |  |  |  |
| Rosaceae | *Laurocerasus caroliniana* (Mill.) M. Roem. |  | 3.4 | 332.58 | 2 | 2 | 12.19 |  |  |  |
| Rosaceae | *Laurocerasus ilicifolia* (Nutt. ex Hook. & Arn.) M. Roem. |  | 1 | 1819.6 | 2 | 1 | 7.92 |  |  |  |
| Rosaceae | **Malus fusca* (Raf.) C.K. Schneid. |  | -11.9 | 8.35 | 2 | 2 | 12.19 |  |  |  |
| Rosaceae | *Mespilus coccinea* (L.) Castigl. |  | -12.2 | 66.67 | 2 | 2 | 7.62 |  |  |  |
| Rosaceae | *Mespilus flava* (Aiton) Dum. Cours. |  | -0.9 |  | 2 | 2 | 4.88 |  |  |  |
| Rosaceae | *Mespilus punctata* (Jacq.) Dum. Cours. |  | -13.5 | 101.8 | 2 | 2 | 6.10 |  |  |  |
| Rosaceae | **Oxyacantha mollis* (Scheele) Lunell |  | -14.6 | 9 | 2 | 2 | 12.19 |  |  |  |
| Rosaceae | *Oxyacantha succulenta* (Schrad. ex Link) Lunell |  | -19.1 | 22.11 | 2 | 2 | 7.92 |  |  |  |
| Rosaceae | **Padus virginiana* (L.) M. Roem. |  | -26.3 | 81.1 | 2 | 2 | 7.92 |  |  |  |
| Rosaceae | **Prunus alleghaniensis* Porter |  | -5.6 | 153.78 | 2 | 2 | 6.10 |  |  |  |
| Rosaceae | **Prunus angustifolia* Marshall |  | -6 | 441.7 | 2 | 2 | 7.92 |  |  |  |
| Rosaceae | **Prunus emarginata* (Douglas) Walp. |  | -9.9 | 49.71 | 2 | 2 | 7.92 |  |  |  |
| Rosaceae | *Prunus hortulana* L.H. Bailey |  | -8.2 |  | 2 | 2 | 6.10 |  |  |  |
| Rosaceae | **Prunus lanata* (Sudw.) Mack. & Bush |  | -18.5 | 539 | 2 | 2 | 10.97 |  |  |  |
| Rosaceae | *Prunus mexicana* S. Watson |  | -8.4 | 463.91 | 2 | 2 | 7.92 |  |  |  |
| Rosaceae | *Prunus munsoniana* W. Wight & Hedrick |  | -5.5 | 252.7 | 2 | 2 | 9.14 |  |  |  |
| Rosaceae | *Prunus myrtifolia* (L.) Urb. |  | 18.9 |  | 2 | 2 | 12.19 |  |  |  |
| Rosaceae | **Prunus pensylvanica* L.f. |  | -28.9 | 45.8 | 2 | 2 | 12.19 |  |  |  |
| Rosaceae | **Prunus serotina* Ehrh. |  | -14.8 | 94.1 | 2 | 2 | 30.48 |  |  |  |
| Rosaceae | *Prunus subcordata* Benth |  | -3.3 | 824.55 | 2 | 2 | 7.92 |  |  |  |
| Rosaceae | *Prunus umbellata* Elliot |  | 4.2 |  | 2 | 2 | 6.10 |  |  |  |
| Rosaceae | **Pyrus decora* (Sarg.) Hyland |  | -25.2 | 3.59 | 2 | 2 | 20.12 |  |  |  |
| Rosaceae | **Sorbus coronaria* (L.) Mill. |  | -8.5 | 32.85 | 2 | 2 | 9.14 |  |  |  |
| Rosaceae | *Sorbus sitchensis* M. Roem. |  | -20.2 | 3.2 | 2 | 2 | 9.10 |  |  |  |
| Rosaceae | *Vauquelinia californica* (Torr.) Sarg. |  | 5.3 |  | 1 | 1 | 6.10 |  |  |  |
| Rubiaceae | *Casasia clusiifolia* (Jacq.) Urb. | 76.505 | 17.4 |  | 2 | 1 | 3.96 |  |  |  |
| Rubiaceae | *Cephalanthus occidentalis* L. |  | -16.6 | 3.21 | (water) | 2 | 14.94 |  |  |  |
| Rubiaceae | *Guettarda elliptica* Sw. |  | 18.8 |  | 2 | 1 | 6.10 |  |  |  |
| Rubiaceae | *Guettarda scabra* (L.) Vent. |  | 18.8 |  | 2 | 1 | 10.06 |  |  |  |
| Rubiaceae | *Hamelia patens* Jacq. |  | 13.6 | 0.07 | 2 | 1 | 4.88 |  |  |  |
| Rubiaceae | *Pinckneya bracteata* (Bartram) Raf. |  | 7.7 |  | 1 | 2 | 7.92 |  |  |  |
| Rubiaceae | *Psychotria nervosa* Sw. |  | 14.1 |  | 2 | 1 | 4.88 |  |  |  |
| Rutaceae | *Amyris balsamifera* L. | 53.366 | 19.4 |  | 2 | 1 | 10.06 |  |  |  |
| Rutaceae | *Amyris elemifera* L. |  | 11.7 | 63.16 | 2 | 1 | 4.88 |  |  |  |
| Rutaceae | *Helietta parvifolia* (A. Gray) Benth. |  | 10.4 | 19.76 | 3 | 1 | 7.01 |  |  |  |
| Rutaceae | *Ptelea baldwinii* (Greene) A.E. Murray |  | 3.4 | 10.43 | 3 | 2 | 4.88 |  |  |  |
| Rutaceae | *Ptelea trifoliata* L. |  | -12 | 11.7 | 3 | 2 | 7.50 |  |  |  |
| Rutaceae | *Zanthoxylum americanum* Mill. |  | -16.4 | 14.3 | 2 | 2 | 10.06 |  |  |  |
| Rutaceae | *Zanthoxylum clava-herculis* L. |  | 2.6 | 28.5 | 2 | 1 | 9.14 |  |  |  |
| Rutaceae | *Zanthoxylum fagara* (L.) Sarg. |  | 10.2 | 12.8 | 2 | 1 | 9.14 |  |  |  |
| Rutaceae | *Zanthoxylum spinosum* (L.) Sw. |  | 18.7 |  | 2 | 1 | 7.01 |  |  |  |
| Salicaeae | **Populus angustifolia* E. James | **58.040** | -14.5 | 2.68 | 3 | 2 | 18.29 | 73 | 55–60 | Boucher *et al*., 2003 |
| Salicaeae | **Populus balsamifera* L. |  | -31.8 | 0.24 | 3 | 2 | 30.48 |  |  |  |
| Salicaeae | **Populus deltoides* W. Bartram ex Marshall |  | -18.4 | 1.15 | 3 | 2 | 30.48 |  |  |  |
| Salicaeae | **Populus fremontii* S. Watson |  | -9 | 0.56 | 3 | 2 | 30.48 |  |  |  |
| Salicaeae | **Populus heterophylla* L. |  | -5.5 | 3.01 | 3 | 2 | 30.48 |  |  |  |
| Salicaeae | *Populus palmeri* Sarg. |  | 6.6 |  | 3 | 2 | 18.29 |  |  |  |
| Salicaeae | **Populus tremula* L. |  | -18.5 | 0.13 | 3 | 2 | 20.12 |  |  |  |
| Salicaeae | **Populus tremuloides* Michx. |  | -31 | 0.1 | 3 | 2 | 18.29 |  |  |  |
| Salicaeae | *Populus trichocarpa* Torr. & A. Gray |  | -19 |  | 3 | 2 | 49.99 |  |  |  |
| Salicaeae | *Salix alaxensis* (Andersson) Coville |  | -34.8 |  | 3 | 2 | 10.06 |  |  |  |
| Salicaeae | **Salix amygdaloides* Andersson |  | -21.5 | 0.13 | 3 | 2 | 20.12 |  |  |  |
| Salicaeae | *Salix arbusculoides* Andersson |  | -32.5 |  | 3 | 2 | 9.14 |  |  |  |
| Salicaeae | **Salix bebbiana* Sarg. |  | -30.6 | 0.18 | 3 | 2 | 7.62 |  |  |  |
| Salicaeae | *Salix bonplandiana* Kunth |  | 2.6 |  | 3 | 2 | 15.24 |  |  |  |
| Salicaeae | **Salix caroliniana* Michx. |  | -4.9 | 0.06 | 3 | 2 | 10.06 |  |  |  |
| Salicaeae | *Salix discolor* (Pursh) Andersson |  | -25.9 | 0.32 | 3 | 2 | 7.92 |  |  |  |
| Salicaeae | *Salix eriocephala* Michx. |  | -21.9 |  | 3 | 2 | 15.24 |  |  |  |
| Salicaeae | *Salix exigua* Nutt. |  | -30.4 | 0.04 | 3 | 2 | 7.92 |  |  |  |
| Salicaeae | *Salix floridana* Chapm. |  | 8.3 |  | 3 | 2 | 7.92 |  |  |  |
| Salicaeae | *Salix gooddingii* C.R .Ball |  | -7.7 | 0.07 | 3 | 2 | 18.29 |  |  |  |
| Salicaeae | *Salix hookeriana* Barratt ex Hook. |  | -13.4 |  | 3 | 2 | 9.14 |  |  |  |
| Salicaeae | *Salix interior* Rowlee |  | -31.5 | 0.041 | 3 | 2 | 6.10 |  |  |  |
| Salicaeae | *Salix laevigata* Bebb |  | -2.8 |  | 3 | 2 | 15.24 |  |  |  |
| Salicaeae | *Salix lasiandra* Benth. |  | -30.2 | 0.04 | 3 | 2 | 18.29 |  |  |  |
| Salicaeae | *Salix lasiolepis* Benth. |  | -8.2 | 0.134 | 3 | 2 | 12.19 |  |  |  |
| Salicaeae | *Salix lucida* Muhl. |  | -27 | 0.173 | 3 | 2 | 7.92 |  |  |  |
| Salicaeae | *Salix melanopsis* Nutt. |  | -0.6 | 0.035 | 3 | 2 | 7.01 |  |  |  |
| Salicaeae | *Salix monticola* Bebb |  | -29.8 |  | 3 | 2 | 6.10 |  |  |  |
| Salicaeae | **Salix nigra* (Pursh) Torr. |  | -14.6 | 0.06 | 3 | 2 | 18.29 |  |  |  |
| Salicaeae | *Salix pellita* (Andersson) Bebb |  | -27 |  | 3 | 2 | 5.00 |  |  |  |
| Salicaeae | *Salix petiolaris* Sm. |  | -27.4 | 0.91 | 3 | 2 | 7.01 |  |  |  |
| Salicaeae | **Salix pyrifolia* Andersson |  | -31.4 |  | 3 | 2 | 7.92 |  |  |  |
| Salicaeae | *Salix rigida* Muhl. |  | -26.2 |  | 3 | 2 | 9.14 |  |  |  |
| Salicaeae | *Salix scouleriana* Barratt ex Hook. |  | -30.1 | 0.07 | 3 | 2 | 7.62 |  |  |  |
| Salicaeae | *Salix sitchensis* Sanson ex Bong. |  | -22 | 0.067 | 3 | 2 | 5.00 |  |  |  |
| Salicaeae | *Salix taxifolia* Kunth |  | -2.9 |  | 3 | 2 | 15.24 |  |  |  |
| Salicaeae | *Salix tracyi* C.R. Ball |  | 2.9 |  | 3 | 2 | 6.10 |  |  |  |
| Sapindaceae | *Acer circinatum* Pursh | **58.745** | -9.6 | 75.7 | 3 | 2 | 14.94 | 51 | 65–99 | Stevens, 2001 |
| Sapindaceae | **Acer glabrum* Torr. |  | -15.6 | 37.4 | 3 | 2 | 8.99 |  |  |  |
| Sapindaceae | *Acer macrophyllum* Pursh |  | -7.7 | 143.1 | 3 | 2 | 29.87 |  |  |  |
| Sapindaceae | **Acer negundo* L. |  | -23 | 38.8 | 3 | 2 | 19.99 |  |  |  |
| Sapindaceae | **Acer pensylvanicum* L. |  | -15.5 | 37.3 | 3 | 2 | 11.89 |  |  |  |
| Sapindaceae | **Acer rubrum* L. |  | -18.4 | 19.3 | 3 | 2 | 28.04 |  |  |  |
| Sapindaceae | **Acer saccharinum* L. |  | -16.9 | 201.5 | 3 | 2 | 29.87 |  |  |  |
| Sapindaceae | **Acer saccharum* Marshall |  | -18.3 | 55.2 | 3 | 2 | 29.87 |  |  |  |
| Sapindaceae | **Acer spicatum* Lam. |  | -23.2 | 20.2 | 3 | 2 | 29.87 |  |  |  |
| Sapindaceae | **Aesculus californica* (Spach) Nutt. |  | -1 | 36756.68 | 1 | 2 | 9.14 |  |  |  |
| Sapindaceae | **Aesculus flava* Sol. |  | -3.7 | 12530.9 | 1 | 2 | 25.91 |  |  |  |
| Sapindaceae | **Aesculus glabra* Willd. |  | -8.5 | 7983.5 | 1 | 2 | 14.94 |  |  |  |
| Sapindaceae | **Aesculus pavia* L. |  | -1.3 | 6516 | 1 | 2 | 6.10 |  |  |  |
| Sapindaceae | **Aesculus sylvatica* W. Bartram |  | 0.9 | 11351.9 | 1 | 2 | 10.06 |  |  |  |
| Sapindaceae | *Dodonaea viscosa* Jacq. |  | 5.8 | 8 | 2 | 1 | 3.05 |  |  |  |
| Sapindaceae | *Exothea paniculata* (Juss.) Radlk. |  | 14.7 |  | 2 | 1 | 14.94 |  |  |  |
| Sapindaceae | *Hypelate trifoliata* Sw. |  | 18.8 |  | 2 | 1 | 13.11 |  |  |  |
| Sapindaceae | *Sapindus drummondii* Hook. & Arn. |  | 9.3 | 575.25 | 2 | 2 | 14.94 |  |  |  |
| Sapindaceae | **Sapindus saponaria* L. |  | -3.2 | 391.1 | 2 | 1 | 14.94 |  |  |  |
| Sapindaceae | *Ungnadia speciosa* Endl. |  | 4 |  |  | 2 | 9.14 |  |  |  |
| Sapotaceae | *Chrysophyllum oliviforme* L. | 93.139 | 15 |  | 2 | 1 | 4.88 | 77 | >73 | Stevens, 2001 |
| Sapotaceae | *Manilkara jaimiqui* (C. Wright ex Griseb.) Dubard |  | 19.1 |  | 2 | 1 | 10.06 |  |  |  |
| Sapotaceae | **Sideroxylon celastrinum* (Kunth) T.D. Penn. |  | 15.5 | 67.72 | 2 | 2 | 6.10 |  |  |  |
| Sapotaceae | *Sideroxylon foetidissimum* Jacq. |  | 14.7 |  | 2 | 1 | 24.99 |  |  |  |
| Sapotaceae | **Sideroxylon lanuginosum* Michx. |  | -2.7 |  | 2 | 2 | 12.19 |  |  |  |
| Sapotaceae | *Sideroxylon lycioides* L. |  | -1.1 |  | 2 | 2 | 9.14 |  |  |  |
| Sapotaceae | *Sideroxylon salicifolium* (L.) Lam. |  | 17.3 |  | 2 | 1 | 16.15 |  |  |  |
| Sapotaceae | *Sideroxylon tenax* L. |  | 7.2 |  | 2 | 2 | 9.14 |  |  |  |
| Schisandraceae | *Illicium floridanum* J. Ellis | 77.026 | 3.7 | 6.1877 | 2 | 1 | 7.92 |  |  |  |
| Schisandraceae | *Illicium parviflorum* Michx. ex Vent. |  | 13.4 | 8.084 | 2 | 1 | 4.57 |  |  |  |
| Simaroubaceae | *Castela emoryi* (A. Gray) Moran & Felger | 49.350 | 6.9 | 34.59 | 1 | 2 | 3.99 |  |  |  |
| Simaroubaceae | *Leitneria floridana* Chapm. |  | 0.8 |  |  | 2 | 7.92 |  |  |  |
| Simaroubaceae | *Simarouba amara* Aubl. |  | 15.5 | 944 | 2 | 1 | 14.94 |  |  |  |
| Solanaceae | *Solanum erianthum* D. Don | 61.878 | 8.2 | 0.66 | 2 | 1 | 4.88 |  |  |  |
| Staphyleaceae | *Staphylea bolanderi* A. Gray | 42.745 | -2.9 | 37.9 | 1 | 2 | 6.10 |  |  |  |
| Staphyleaceae | *Staphylea trifolia* L. |  | -13.2 | 41.53 |  | 2 | 7.92 |  |  |  |
| Styracaceae | **Carlomohria parviflora* (Michx.) Greene | 60.784 | 5.9 |  | 1 | 2 | 9.14 | **54** | 50–56 | Tiffney, 1985 |
| Styracaceae | **Halesia carolina* L. |  | -1.7 | 277.78 | 1 | 2 | 12.19 |  |  |  |
| Styracaceae | **Halesia diptera* L. |  | 3.5 |  | 3 | 2 | 10.06 |  |  |  |
| Styracaceae | *Styrax grandifolius* Aiton |  | -2.3 | 56.3 | 1 | 2 | 12.19 |  |  |  |
| Surianaceae | *Suriana maritima* L. | 63.804 | 15.4 | 11.76 | (water) | 1 | 4.88 |  |  |  |
| Symplocaceae | *Symplocos tinctoria* (L.) L'Hér. | 82.167 | -0.2 |  | 2 | 1 | 10.06 |  |  |  |
| Theaceae | **Gordonia lasianthus* (L.) Ellis | 82.167 | 5 | 3.51 | 3 | 1 | 20.12 | 77 | 45–99 | Grote & Dilcher, 1989 |
| Theaceae | *Stewartia malacodendron* L. |  | -0.8 |  | 2 | 2 | 6.10 |  |  |  |
| Theaceae | *Stewartia ovata* (Cav.) Weath. |  | -0.5 |  | 3 | 2 | 6.10 |  |  |  |
| Ulmaceae | **Planera aquatica* J.F. Gmel. | 52.390 | -0.7 |  | (water) | 2 | 15.24 | **66** | 65–70 | Manchester, 1989 |
| Ulmaceae | **Ulmus alata* Michx. |  | -2.3 | 4 | 3 | 2 | 20.12 |  |  |  |
| Ulmaceae | **Ulmus americana* L. |  | -22.2 | 5.4 | 3 | 2 | 40.23 |  |  |  |
| Ulmaceae | **Ulmus crassifolia* Nutt. |  | 1.1 | 6.27 | 3 | 2 | 20.12 |  |  |  |
| Ulmaceae | **Ulmus rubra* Muhl. |  | -16 | 11.08 | 3 | 2 | 24.99 |  |  |  |
| Ulmaceae | **Ulmus serotina* Sarg. |  | -2.1 | 3.02 | 3 | 2 | 20.12 |  |  |  |
| Ulmaceae | **Ulmus thomasii* Sarg. |  | -16.1 | 60.5 | 3 | 2 | 35.05 |  |  |  |
| Verbenaceae | **Avicennia germinans* (L.) L. | 39.728 | 7.2 | 10120 | (water) | 1 | 11.89 | 36 | --- | --- |
| Verbenaceae | *Citharexylum spinosum* L. |  | 15.5 | 135.63 | 2 | 1 | 11.89 |  |  |  |
| Zygophyllaceae | *Guaiacum angustifolium* Engelm. | 94.241 | 6.8 | 126.83 | 2 | 1 | 6.10 |  |  |  |
| Zygophyllaceae | *Guaiacum sanctum* L. |  | 19.6 | 333 | 2 | 1 | 10.06 |  |  |  |

**Note:** Dispersal type codes are 1 = unassisted/short distance, 2 = animal/medium distance, and 3 = wind/long distance; Leaf phenology codes are 1 = evergreen and 2 = deciduous.

**References**

Angiosperm Phylogeny Group (2009) An update of the Angiosperm Phylogeny Group classification for the orders and families of flowering plants: APG III. *Botanical Journal of the Linnean Society*, **161**, 105–121

Basinger, J. F. & Christophel, D. C. (1985) Fossil flowers and leaves of the Ebenaceae from the Eocene of southern Australia. *Canadian Journal of Botany*, **63**, 1825–1843.

Bell, C. D., Soltis, D. E. & Soltis, P. S. (2010) The age and diversification of the angiosperms rerevisited. *American Journal of Botany*, **97**, 1296–1303

Biffin, E., Lucas, E. J., Craven, L. A., Ribeiro da Costa, I., Harrington, M. G. & Crisp, M. D. (2010) Evolution of exceptional species richness among lineages of fleshy-fruited Myrtaceae. *Annals of Botany*, **106**, 79–93.

Boucher, L. D., Manchester, S. R. & Judd, W. S. (2003) An extinct genus of Salicaceae based on twigs with attached flower, fruits, and foliage from the Eocene Green River Formation of Utah and Colorado, USA. *American Journal of Botany*, **90**, 1389–1399.

Calvillo-Candell, L., & Cevallos-Ferriz, S. R. S. (2007) Reproductive structures of Rhamnaceae from the Cerro del Pueblo (Late Cretaceous, Coahuila) and Coatzingo (Oligocene, Puebla) Formations, Mexico. *American Journal of Botany*, **94**, 1658–1669.

Cuénoud, P., Martinez, M. A. P., Loizeau, P.-A., Spichiger, R., Andrews, S. & Manen, J.-F. (2000) Molecular phylogeny and biogeography of the genus *Ilex* L. (Aquifoliaceae). *Annals of Botany*, **85**, 111–122.

Davies, T. J., Barraclough, T. G., Chase, M. W., Soltis, P. S., Soltis, D. E. & Savolainen, V. (2004) Darwin’s abominable mystery: insights from a supertree of the angiosperms. *Proceedings of the National Academy of Sciences USA*, **101**, 1904–1909.

DeVore, M. L., & Pigg, K. B. (2007) A brief review of the fossil history of the family Rosaceae with a focus on the Eocene Okanogan Highlands of eastern Washington State, USA, and British Columbia, Canada. *Plant Systematics and Evolution*, **266**, 45–57.

Drinnan, A. N., Crane, P. R., Friis, E. M. & Pedersen, K. R. (1990) Lauraceous flowers from the Potomac Group (mid-Cretaceous) of eastern North America. *Botanical Gazette*, **151**, 370–384.

Forest, F., Savolainen, V., Chase, M. W., Lupia, R., Bruneau, A. & Crane, P. R. (2005) Teasing apart molecular- versus fossil-based error estimates when dating phylogenetic trees: a case study in the birch family (Betulaceae). *Systematic Botany*, **30**, 118–133.

Friis, E. M., Crane, P. R. & Pedersen, K. R. (2011) *Early flowers and angiosperm evolution*. Cambridge University Press, Cambridge, UK.

Graham. A. (2006) Paleobotanical evidence and molecular data in reconstructing the historical phytogeography of Rhizophoraceae. *Annals of the Missouri Botanical Garden*, **93**, 325–334.

Grote, P. J. & Dilcher, D. L. (1989) Investigations of angiosperms from the Eocene of North America: a new genus of Theaceae based on fruit and seed remains. *Botanical Gazette*, **150**, 190–206.

Harly, M. M. (2006) A summary of fossil records for Arecaceae. *Botanical Journal of the Linnean Society*, **151**, 39–67.

Herendeen, P. S., Crepet, W. L. & Dilcher, D. L. (1992) The fossil history of the Leguminosae: phylogenetic and biogeographic implications. *Advances in legume systematics, part 4, the fossil record* (eds P. S. Herendeen & D. L. Dilcher), pp. 303–316. Royal Botanic Gardens, Kew.

Hickey, L. J., & Doyle, J. A. (1977) Early Cretaceous fossil evidence for angiosperm evolution. *Botanical Review*, **43**, 2–104.

Hinsley, S. R. (2009) Malvaceae info [http://www.malvaceae.info/index.html, accessed November, 2012].

Jordan, G. J., Bannister, J. M., Mildenhall, D. C., Zetter, R. & Lee, D. E. (2009) Fossil Ericacee from New Zealand: deconstructing the use of fossil evidence in historical biogeography. *American Journal of Botany*, **97**, 59–70.

Manchester, S. R. (1989) Systematics and fossil history of the Ulmaceae. *Evolution, systematics, and fossil history of the Hamamelidae* (eds P. R. Crane & S. Blackmore), pp. 221–251. Clarendon Press, Oxford.

Manos, P. S., Soltis, P. S., Soltis, D. E., Manchester, S. R., Oh, S.-H., Bell, C. D., Dilcher, D. L. & Stone, D. E. (2007) Phylogeny of extant and fossil Juglandaceae inferred from the integration of molecular and morphological data sets. *Systematic Biology*, **56**, 412–430.

Miller, J. S. (2002) A revision of *Ehretia* (Boraginaceae) for Madagascar and the Comoro Islands. *Adansonia*, **24**, 137–157.

Muellner, A. N., Savolainen, V., Samuel, R. & Chase, M. W. (2006) The mahogany family “out-of-Africa”: divergence time estimation, global biogeographic patterns inferred from plastid *rbcL* DNA sequences, extant, and fossil distribution of diversity. *Molecular Phylogenetics and Evolution*, **40**, 236–250.

Muller, J. (1981) Fossil pollen records for extant angiosperms. *Botanical Review*, **47**, 1–142.

Pell, S. K. (1995) Molecular systematics of the cashew family (Anacardiaceae). Ph.D. dissertation, Louisiana State University, Baton Rouge, LA.

Pirie, M. D. & Doyle, J. A. (2012) Dating clades with fossils and molecules: the case of Annonaceae. *Botanical Journal of the Linnean Society*, **169**, 84–116.

Stevens, P. F. (2001 onwards) Angiosperm phylogeny website. Version 12, July 2012, http://www.mobot.org/MOBOT/research/APweb/.

Taylor, T. N., Taylor, E. L. & Krings, M. (1993) *Paleobotany: the biology and evolution of fossil plants*. Prentice Hall, Englewood Cliffs, NJ.

Tiffney, B. H. (1985) The Eocene North Atlantic land bridge: its importance in Tertiary and modern phytogeography of the Northern Hemisphere. *Journal of the Arnold Arboretum*, **66**, 243–273.

Weeks, A., Daly, D. C. & Simpson, B. B. (2005) The phylogenetic history and biogeography of the frankincense and myrrh family (Burseraceae) based on nuclear and chloroplast sequence data. *Molecular Phylogenetics and Evolution*, **35**, 85–101.

Wehr, W. C. & Hopkins, D. Q. (1994) The Eocene orchards and gardens of Republic, Washington. *Washington Geology*, **22[September]**:27–34.

Zerega, N. J. C., Clement, W. L., Datwyler, S. L. & Weiblen, G. D. (2005) Biogeography and divergence times in the mulberry family (Moraceae). *Molecular Phylogenetics and Evolution*, **37**, 402–416.

Zhou, Z.-K., Crepet, W. L. & Nixon, K. C. (2001) The earliest fossil evidence of the Hamamelidaceae: Late Cretaceous (Turonian) inflorescences and fruits of Altingioideae. *American Journal of Botany*, **88**, 753–766.
